# Monocationic versus dicationic-based monomethine cyanine dyes for ultrasensitive colorimetric detection of hypochlorite ion in water

**DOI:** 10.1038/s41598-025-88839-y

**Published:** 2025-02-15

**Authors:** Nermeen S. Hafez, Wael A. Amer, Ehab A. Okba, Mahmoud A. S. Sakr, Hussein H. Alganzory, Sohaila M. Khalil, El-Zeiny M. Ebeid

**Affiliations:** 1 Chemistry Department, Faculty of Science, Tanta University, Tanta, 31527 Egypt; 2 Chemistry Department, College of Science, Bahrain University, Sakhir, 32038 Bahrain; 3 Center of Basic Science, Misr University for Science and Technology, 6th of October City, Egypt; 4 Chemistry Department, Faculty of Science, Benha University, Benha, Egypt; 5 Immunology and Parasitology Division, Zoology Department, Faculty of Science, Center of Excellence in Cancer Research, New Tanta University Teaching Hospital, Tanta University, Tanta, Egypt

**Keywords:** Cyanine hypochlorite sensors, Naked-eye hypochlorite detection, Tap water, Quartz crystal microbalance, Radical cation formation, Quantum chemical studies, Chemistry, Optics and photonics

## Abstract

Detecting residual chlorine as a hypochlorite ion (ClO^−^) in drinking water is crucial for ensuring disinfection effectiveness and safety. In the present study, we report two novel Quinolium Benzothiazole-Based Cyanine (3ethylbenzothiazol-2(3 H)-ylidene)methyl)-1-(4-iodobutyl)quinolin-1-ium tetrafluoroborate (IBTQ) and 1-(3-(4-(dimethylamino)pyridin-1-ium-1-yl)propyl)-4-((3-methylbenzothiazol-2(3 H)-ylidene)methyl)quinolin-1-ium diiodide (DMP-BTQ) hypochlorite (ClO^−^) sensors using UV- visible, colorimetric, and quartz crystal microbalance (QCM) techniques. The two sensors generate distinct absorption spectra, frequency shifts, and color changes that are visible to the naked eye. They exhibit high sensitivity and selectivity towards ClO^−^. The sensors have limits of detection (LOD) values in the range of 13.92 ppm and 0.127 ppm for IBTQ and DMP-BTQ, respectively, based on absorption performance with no interference of potential ions in drinking water. The method yields good recovery results, ranging from 97.4 to 103.0%, for ClO^−^ detection in the studied water samples. In addition, the LOD for the QCM technique is 0.06 ppm for IBTQ and 0.045 ppm for DMP-BTQ with low quantification. The sensors can be loaded on paper strips for naked-eye detection of ClO^−^ in domestic tap water and water treatment facilities. The sensors also provide low-cost, low cytotoxicity, high sensitivity, selectivity, and reusability of ClO^−^ in water. The sensing mechanism was rationalized in terms of radical cation generation upon ClO^−^ oxidizing action. The ease of cyanine oxidation was substantiated by quantum chemical studies including density functional theory (DFT) calculations, natural bond orbital (NBO) analysis, molecular electrostatic potential (MESP), and time-dependent density functional theory to support the experimental results.

**Supplementary Information:**

The online version contains supplementary material available at 10.1038/s41598-025-88839-y.

## Introduction

Hypochlorous acid (HOCl) is a potent oxidizer used in household bleaches and disinfectants for its antibacterial and stain-removing properties. It reacts quickly with organic and inorganic compounds, making it essential for residential and industrial cleaning. HOCl’s disinfection properties extend to medical, food, and water treatment sectors, protecting public health and food safety^1^. However, excessive exposure can lead to severe health consequences, such as lipid peroxidation^2^, cellular membrane integrity^3^, protein denaturation^4^, and DNA strand breaks^5^, potentially leading to diseases like arthritis^6^, cardiovascular disorders^7^, kidney ailments^8^, neurodegenerative conditions^9^, and cancer^10^. The World Health Organization recommends limiting chlorine in drinking water^11^. So, the detection of hypochlorite ions in water is crucial for disinfection processes and sanitation systems. Various analytical techniques, including spectrophotometry^12^, chemiluminescence^13^, and colorimetric approaches^14^ have been explored but often face limitations like low sensitivity and selectivity. Recent developments in colorimetric probes for hypochlorite ions (ClO^-^) have led to advances, but still have limitations^15^. Tricyanoethylene-derived colorimetric probes, which use tricyanovinyl dyes as chromophores, offer a simple, fast, and water-soluble solution^16^. The commonly used colorimetric procedure for measuring chlorine is N, N′-diethyl-p-phenylenediamine, but it can be costly and hazardous^17^. Fluorescent probes have gained popularity due to their high specificity and sensitivity. The Schiff base moiety is a desirable functional group due to its improved sensitivity, selectivity, and fluorescent properties, making it ideal for detecting heavy metal ions, thiols, and reactive oxygen species^18,19^. Cyanine dyes offer advantages like longer wavelengths, high absorption coefficients, fluorescence quantum yield, biocompatibility, and minimal cytotoxicity, making them useful for detecting various ions, nanoparticles, nucleic acids, and biological macromolecules^20^. In this work, we synthesized (3ethylbenzothiazol-2(3 H)-ylidene)methyl)-1-(4-iodobutyl)quinolin-1-ium tetrafluoroborate (IBTQ) and 1-(3-(4-(dimethylamino)pyridin-1-ium-1-yl)propyl)-4-((3-methylbenzothiazol-2(3 H)-ylidene)methyl)quinolin-1-ium diiodide (DMP-BTQ) using a solvent-free microwave-assisted method, resulting in ultra-sensitive sensors capable of detecting trace levels of hypochlorite ions. The study investigates the development of cyanine dyes as sensitive and selective sensors for detecting hypochlorite ions in water samples with a lower detection limit^21–23^.

**Fig. 1:**
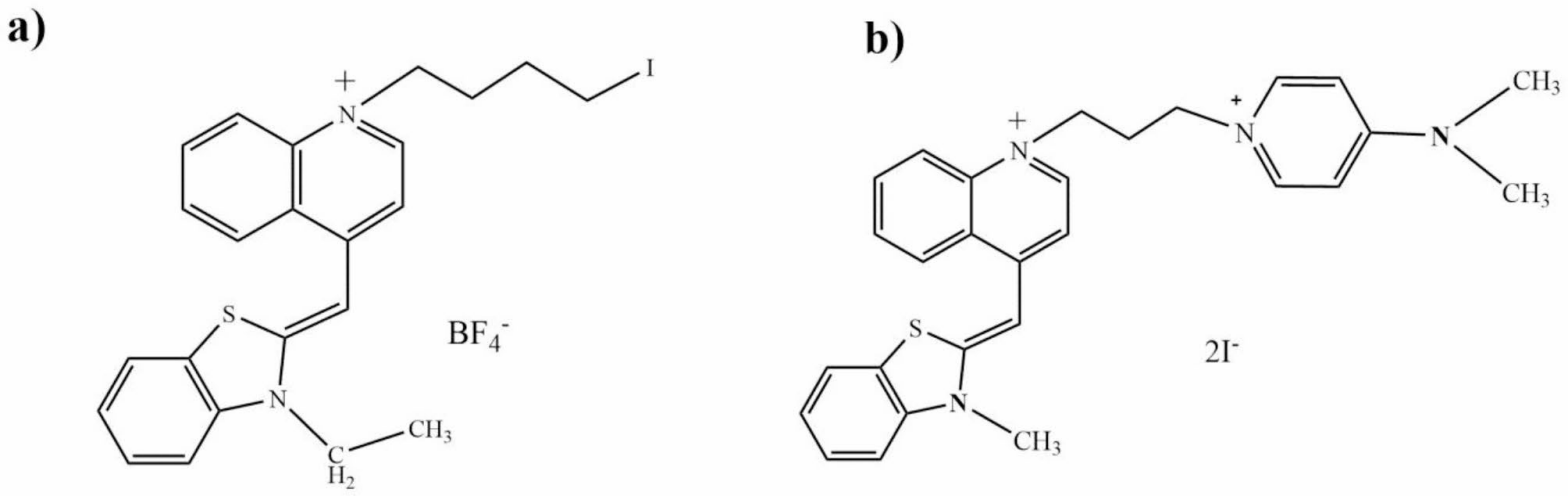
Chemical structures of cyanine dyes (**a**) IBTQ and (**b**) DMP-BTQ.

The study utilizes spectrophotometry and quartz crystal microbalance (QCM) techniques to quantify hypochlorite ion concentrations in water samples using microwave-assisted solvent-free synthesized cyanine dyes (Fig. 1). The QCM methodology was found to be particularly effective for quick and sensitive detection with a low detection limit^24–26^. The mechanism of action for these sensors is based on a radical cation mechanism similar to other hypochlorite sensors, such as 2,2′-azino-bis(3-ethyl-benzothiazoline-6-sulfonic acid) (ABTS) and promethazine hydrochloride sensors^27,28^. Quantum mechanical calculations were performed to confirm the radical pathway mechanism.

The study emphasizes the benefits of using cyanine dyes as hypochlorite sensors, including their high sensitivity and selectivity, low cytotoxicity, as well as their applicability in colorimetric detection method. Previous studies have also demonstrated the effectiveness of cyanine dyes in detecting hypochlorite levels in drinking water.

In summary, the study presents the development of cyanine dyes as sensitive and selective sensors for detecting hypochlorite ions in water samples, which were synthesized using a solvent-free microwave-assisted method with highly sensitive and selective critera. The study employs spectrophotometry and QCM techniques to quantify hypochlorite ion concentrations in water samples, and quantum mechanical calculations were performed to confirm the radical pathway mechanism.

## Experimental

### Synthesis of cyanine sensors

Cyanine sensors were prepared by microwave-assisted synthesis. For IBTQ, equivalent amounts of 3-ethyl-2-(methylthio)-3a,7a-dihydrobenzo[d]thiazol-3-ium tetrafluoroborate 0.906 gm (2 mmol) and 1-(4-iodobutyl)-4-methylquinolin-1-ium iodide 0.594 gm (2 mmol) were mixed in a glass conical flask. A few drops of trimethylamine (1 mL) were also used during this step as a catalyst. The mixture was subjected to microwave irradiation for 6 min at a power of 280 W. After cooling and washing with diethyl ether, an orange to yellowish-orange precipitate was obtained (Fig. S1a).

The synthesis of DMP-BTQ was performed in two steps. In the first one, a monomethine cyanine dye (1-(3-iodopropyl)-4-((3-methylbenzothiazol-2(3 H)-ylidene)methyl)quinolin-1-ium) was prepared according to literature^29^. In the second step, equivalent amounts of monomethine cyanine dye, the product from the first step, 0.586 gm (1 mmol), and N, N-dimethylpyridin-4-amine 0.122 gm (1 mmol) were stirred in the presence of DMF (20 mL). A little catalytic amount of trimethylamine (1 mL) was also used during this step. The mixture was subjected to microwave irradiation with stirring for 90 min and 100 W power. The formed precipitate was filtered off, washed with CH₂Cl₂, and dried at 60 °C (Fig. S1b). The structures of synthesized sensors were characterized using^1^H NMR (Fig. S12 and S13)^13^, C NMR spectra (Fig. S14), EI-mass (Fig. S15 and S16), FT-IR spectra (Fig. S17), and elemental analyses.

## Results and discussions

### Absorption spectra of IBTQ and DMP-BTQ for hypochlorite detection

**Fig. 2:**
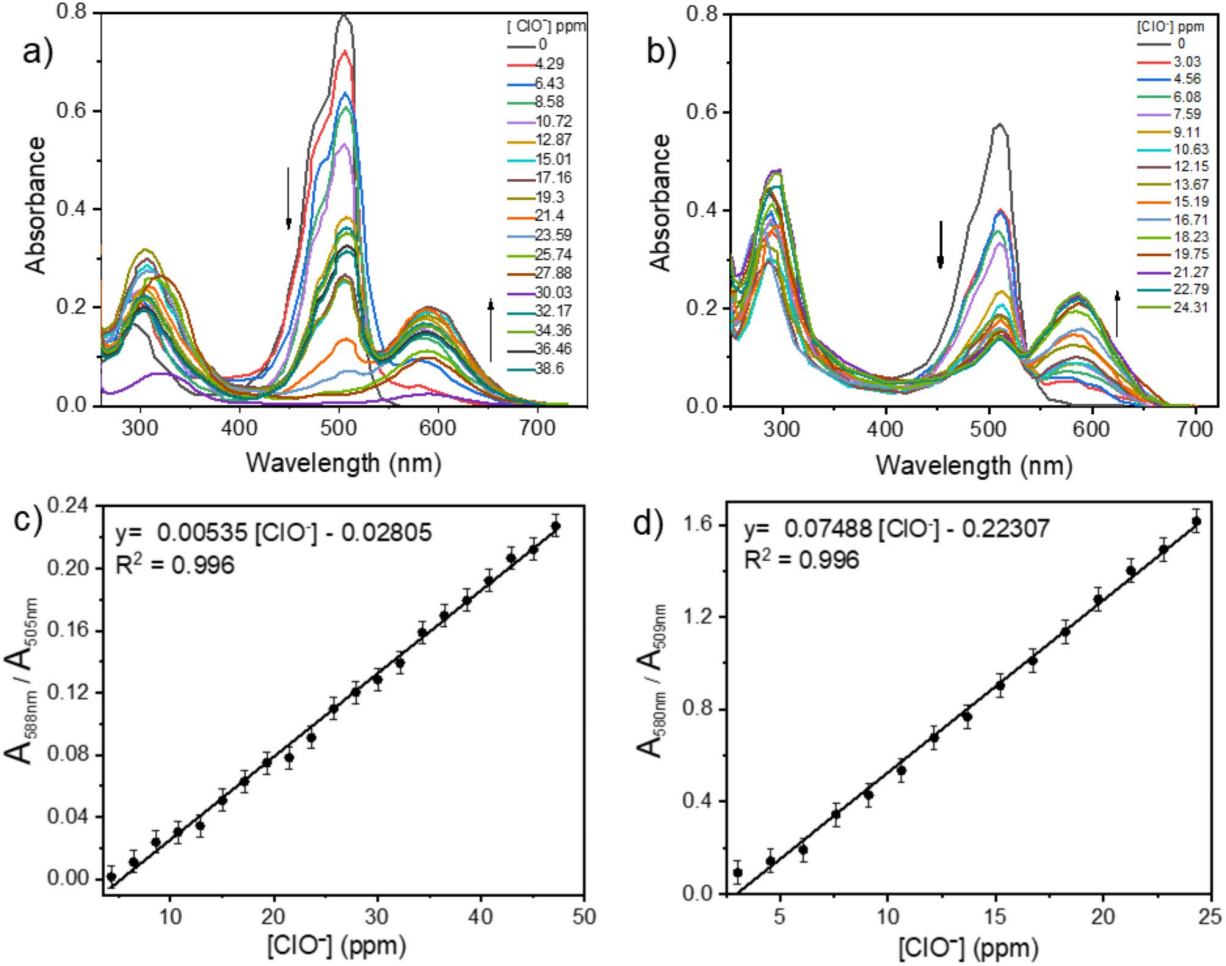
UV–Vis. spectra of (**a**) 15 µM IBTQ and (**b**) 10 µM DMP-BTQ in ethanolic/PBS (10 mM, pH = 7.4) solutions in the presence of varying amounts of ClO^-^. Absorbance ratios of (**c**) IBTQ’s A_588_/A_505_ and (**d**) DMP-BTQ’s A_580_/A_509_ against ClO^-^ concentrations.

Figure 2a, b depicts the electronic absorption of IBTQ (15 µM) and DMP-BTQ (10 µM) in ethanolic /phosphate saline buffer solution (PBS) (10 mM, pH = 7.4), in the presence and absence of hypochlorite ions. As the concentration of ClO^-^ increases, the absorption maxima of IBTQ and DMP-BTQ at 505 nm and 509 nm, respectively, drop progressively. As the concentration of hypochlorite increases, the color of IBTQ and DMP-BTQ gradually changes from red to blue, and the color of the sensor disappears when the concentration of hypochlorite exceeds 0.02 M. As seen in Fig. S22, in the presence of varying ClO^-^ concentrations, IBTQ and DMP-BTQ showed the sensor’s color progressive color shift from red to blue. Furthermore, Fig. 2b shows an isosbestic point at 535 nm, which could be associated with the creation of a single product.

The reaction with hypochlorite ions (ClO^−^) causes oxidation of the sensor molecules, leading to the formation of a radical cation intermediate and subsequently a new molecular structure. The final violet-colored form likely has an extended or altered π-conjugation system compared to the initial red form. This change in conjugation affects the energy levels of the molecular orbitals. The shift from red (absorbing around 500 nm) to violet (absorbing around 580 nm) indicates a reduction in the energy gap between the highest occupied molecular orbital (HOMO) and the lowest unoccupied molecular orbital (LUMO). The new structure may facilitate intramolecular charge transfer, which often results in longer wavelength absorptions. The radical cation formation and subsequent structural changes likely lead to greater electron delocalization across the molecule, which typically results in a bathochromic shift.

UV-visible titration tests were performed by plotting the absorbances A_588nm_/A_505nm_ ratios for IBTQ and A_580nm_/A_509nm_ for DMP-BTQ against the concentration of hypochlorite ions. Figure 2 (c and d) illustrates high correlation coefficients and good linearity for IBTQ (R^2^ = 0.996 in the 4.3–47.18 ppm range) and (R^2^ = 0.996 in 3.03–24.31 ppm range) for DMP-BTQ. These results demonstrate that hypochlorite may be detected calorimetrically using IBTQ and DMP-BTQ in the visible light region. The limits of detection (LOD) of ClO^−^ IBTQ and DMP-BTQ were derived using the formula LOD = 3σ/m, where σ is the standard deviation of the blank measurements and m is the slope of the calibration curve. The LOD values for IBTQ and DMP-BTQ are 13.92 ppm and 0.127 ppm, respectively. The limits of quantification (LOQ) for ClO^-^ were determined using the formula LOQ = 10σ/m, where σ is the standard deviation of the blank measurements and m is the slope of a calibration curve^30^. The LOQ values were calculated as 45.93 ppm and 0.425 ppm for IBTQ and DMP-BTQ, respectively. The sensors’ LOD values are below the WHO-set permissible level for drinking water, suggesting that sensors could be a useful tool for quantitatively detecting HOCl / OCl^-^ in aqueous conditions^31^. Under physiological settings, the current sensors can thus detect HOCl / OCl^-^ for practical detection. The LOD values produced from the relationship 3σ/m are essentially beyond the instrumental sensitivity available. We did real-world testing with the smallest concentrations of ClO^-^ capable of producing detectable signals on the Shimadzu-50 UV-Vis. spectrophotometer. The experimentally discernible limitations are shown in Table S1.

### Selectivity of IBTQ and DMP-BTQ for hypochlorite ions

**Fig. 3:**
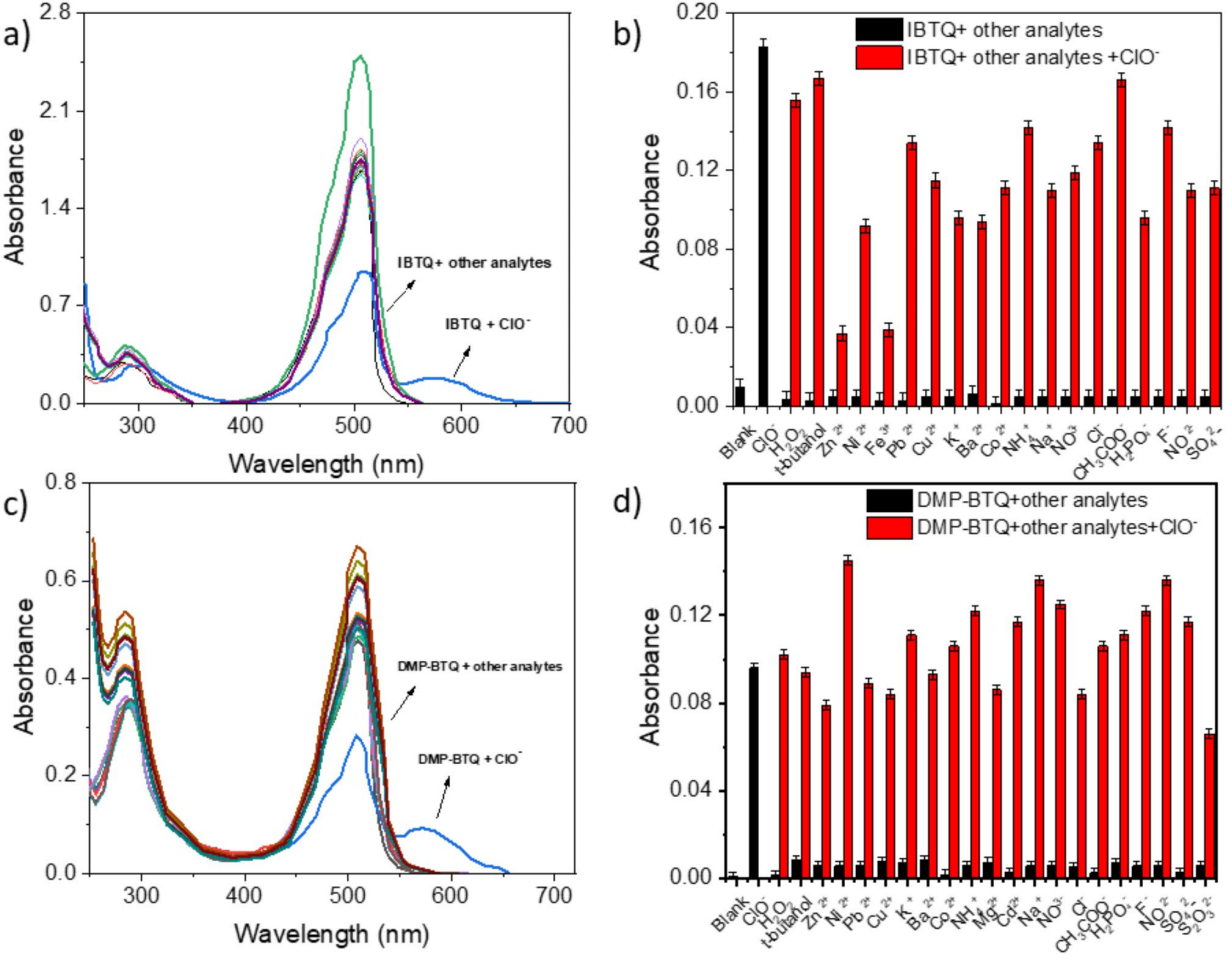
(**a**) Absorption spectra and (**b**) Absorbances of IBTQ (15 µM) at 588 nm with various analytes (35 ppm); (**c**) Absorption spectra and (**d**) absorbances of DMP-BTQ (10 µM) at 580 nm in absence and presence of various analytes (5 ppm).

IBTQ and DMP-BTQ showed high selectivity for ClO^–^ over other analytes, such as Zn^2+^, Ni^2+^, Co^2+^, Ba^2+^, K^+^, Fe^3+^, Pb^2^, NH_4_^+^, Cu^2+^, Na^+^, Cl^−^, F^−^, NO_3_^−^, NO_2_^−^, CH_3_COO^−^, SO_4_^2−^, H_2_PO_4_^−^, t-butanol and H_2_O_2_ (Fig. 3). Among the ions tested, only ClO^−^ generated a change in the UV-Vis. spectra with developed bands at 588 nm for IBTQ (Fig. 3a) and at 580 nm for DMP-BTQ (Fig. 3c). The solution color changed from red to blue, and the color of other analytes did not change. In the selectivity and competition experiment, the presence of these anions did not interfere with ClO^−^ as shown in Fig. 3 (b, and d). The sensors exhibit high selectivity and anti-interference for detecting ClO^−^.

A comparison of LOD values of IBTQ and DMP-BTQ with those of some previously reported HOCl/OCl^−^ probes in literature is given in Table S2. The sensors used in the study are more sensitive and selective, with a lower detection limit as low as 13.92 ppm and 0.127 ppm for the two sensors in the study^21–23,32,33^. This high sensitivity allows for the detection of trace amounts of ClO^−^ in water samples. These results showed that sensors could be used as sensitive colorimetric sensors for the quantitative detection of ClO^−^ with a low LOD compared to other sensors. *N*,* N*-diethyl-*p*-phenylenediamine (DPD), and o-tolidine (OT) are two colorimetric reagents that have been widely used^34,35^. Particularly, DPD has replaced OT as the preeminent alternative due to the latter’s identification as a probable carcinogen^36^.The sensitivity of the DPD method, which is a standard national method, is as low as 0.05 g/mL. However, this technique can be costly and hazardous due to the presence of ethylenedinitrilotetraacetic acid disodium salt dehydrate^17^. This method also showed interference with metal ions, chloramines, organic compounds, and oxidants^37^. 2,2-azino-bis(3-ethylbenzothiazoline)-6-sulfonic acid-diammonium salt (ABTS) is a colorimetric sensor for ClO^−^ and showed interference with bromine and brominated organic compounds^28^. Nitrogen and sulfur co-doped carbon dots (N, S-CDs) showed interference with Fe^3+ 38^ The sensors exhibit high selectivity and anti-interference for detecting ClO^−^ compared with these sensors.

### Quartz crystal microbalance (QCM) measurements

The QCM method was used to examine IBTQ and DMP-BTQ’s suitability as ClO^-^ sensors. The QCM probe-coated electrode was first soaked in DI water until stabilization. Using the Sauerbrey equation (Eq. 1), the mass distribution of cyanine film onto the QCM electrode was calculated to be 8.745 g cm^-2^ and 7.491 g cm^-2^ for IBTQ and DMP-BTQ, respectively. The QCM’s frequency (*f*) was obtained as a function of time.

The sensors undergo oxidation through a process that involves a single electron transfer, forming a radical cation, and then oxidation, leading to the breakdown of the sensor molecule. This process is facilitated by forming radical cations, which are more soluble and become leached from the electrode surface. Hypochlorite is a stronger oxidizing agent (E° = +0.89 V vs. SHE). Hypochlorite’s faster reaction with the sensors is likely due to its higher redox potential and the reactive nature of the benzothiazole quinolinium system. This strong oxidizing nature and the formation of radical cations explain the observed frequency increase in the QCM study, as the breakdown of sensor molecules into smaller, soluble compounds results in mass loss from the QCM surface (Fig. S2 a and c). As the concentration of ClO^−^ increased, the frequency increases. The association between ClO^−^ concentrations and QCM (∆*f*) response is linear as shown in Fig. S2b. A calibration curve was constructed for IBTQ showing a good correlation with high linearity [y = 8.10337 [ClO^−^] + 16.51818] in the concentration range 0.416–1.53 ppm. A calibration curve was constructed for DMP-BTQ, and a linear correlation was found [y = 104.74229 [ClO^−^] – 7.9214] with good linearity in the range of 0.142 to 0.68 ppm (Fig. S2d). The LOD values were determined using the formula LOD = 3.3σ/m, where σ is the y-intercept standard deviation of a linear correlation and m is the slope^38^. The detection limits were determined to be 0.06 ppm for IBTQ and 0.045 ppm for DMP-BTQ. The LOQ values were calculated using the formula LOQ = 10 σ/m, where σ is the y-intercept standard deviation of a linear correlation and m is the slope of the linear correlation^38^. The LOQs were calculated to be 0.182 ppm for IBTQ and 0.136 ppm for DMP-BTQ. All these findings suggest that the QCM approach can be applied to study cyanine sensors’ action on ClO^−^.

### Response time and pH-dependence for hypochlorite detection

Time-dependence of IBTQ and DMP-BTQ absorbance change upon ClO^-^ action was examined. According to Fig. S3 (a and b), sensor’s IBTQ and DMP-BTQ absorbances at 588 nm and 580 nm steadily decrease and a plateau is attained in 7 min.

The stopped-flow technique demonstrates rapid reactions between sensors and hypochlorite, showing significant changes within 30 s (Fig.S4). It captures fast kinetics missed by slower mixing techniques. The technique monitors fast chemical processes in real-time, providing insights into sensors’ performance in the rapid detection of hypochlorite. In addition, the absorbance of the sensors had no obvious change after storage for 192 h, indicating long-term stability for practical applications (Fig. S5).

The performance of the examined cyanine sensors was significantly influenced by the pH of the medium. The influence of PBS pH on both sensors’ absorption behavior was examined to ascertain whether sensors respond to ClO^-^ under physiological conditions. As could be seen in Fig. S3 (c and d), neither of the sensors under investigation underwent a noticeable change in absorption intensity before the addition of ClO^-^. The absorbance for IBTQ at 588 nm increased as the pH value increased until a plateau was attained in the pH range 6–11 upon ClO^–^ addition. For DMP-BTQ, the absorbance at 580 nm increased as the value of pH increased until a plateau was attained in the pH range 7–11 upon ClO^–^ addition indicating that DMP-BTQ could be used under physiological conditions.

The stability of the sensors has been studied in different polar solvents (Fig. S6), and the study reveals that IBTQ and DMP-BTQ are most stable in water. They are also more stable in ethanol, acetonitrile, and dioxane. The absorbance decreases as solvent polarity decreases, suggesting they are more stable in polar solvents. IBTQ appears more stable across all solvents and sensitive to solvent changes. These differences may be due to molecular structure differences, affecting solubility and stability in different solvents.

### Concentration effect of IBTQ and DMP-BTQ

The absorbance of IBTQ at 588 nm increased linearly with increasing concentration in the range (0.1–0.7 µM) while for DMP-BTQ, the absorbance increased linearly in the range (15–35 µM) as shown in Fig. S7. Considering the sensitivity and linear range of the technique, final sensor concentrations of 0.7 µM and 35 µM were chosen for IBTQ and DMP-BTQ, respectively.

### Practical application of sensors in water samples

Tap water samples were measured directly without further treatment. Certain amounts of ClO^-^ were added to ethanolic solutions of both sensors in tap water. After mixing thoroughly, the UV-Vis. absorption spectra were measured. The recovery of ClO^-^ was determined from the absorption data of IBTQ and DMP-BTQ as shown in Table 1. The average recovery rate of ClO^-^ in real water samples was about 97.4 ~ 103% with excellent analytical precision (< 4%) in detecting ClO^-^ spiked water samples, indicating that IBTQ and DMP-BTQ are effective ClO^-^ detectors in the real water samples compared with other sensors^20,39–41^. The actual content of ClO^-^ in real water samples was evaluated using a standard national method based on the *N*,* N*-diethyl-*p*-phenylenediamine (DPD) reagent and the results are compared with IBTQ and DMP-BTQ data as shown in Table S3 and Fig. S8. This will aid in validating the sensor’s accuracy^42^.

**Table 1:** Validation for IBTQ and DMP-BTQ using absorption spectroscopy.

Real water samples	ClO^−^ spiked (ppm)	ClO^−^ recovered (ppm)_a_	Recovery (%)	*R*.S.D (%) *N* = 3
Validation for IBTQ				
KZ tap water	2.86	2.83 ± 1.319	99	0.538
	5.72	5.77 ± 1.319	101	0.496
Tanta tap water	2.86	2.93 ± 1.319	102	2.59
	5.72	5.66 ± 1.319	99	1.88
Distilled water	2.86	2.83 ± 1.319	99	2.59
	5.72	5.77 ± 1.319	101	1.844
Bottled water	2.86	2.94 ± 1.319	103	2.71
	5.72	5.66 ± 1.319	99	1.947
Validation for DMP-BTQ				
KZ tap water	1.57	1.53 ± 0.453	97.5	0.349
	2.57	2.54 ± 0.453	99	0.33
Tanta tap water	1.57	1.53 ± 0.45	97.4	3.85
	2.57	2.54 ± 0.453	99	2.41
Distilled water	1.57	1.54 ± 0.45	97.4	3.85
	2.57	2.55 ± 0.44	99.2	2.4
Bottled water	1.57	1.54 ± 0.4	97.4	3.8
	2.57	2.55 ± 0.43	99.2	2.41

^a^Mean ± standard deviation (*n* = 3).

### Proposed detection mechanism

The probes for HOCl can be categorized into different types based on their reaction sites: oxidative cleavage of C=C bonds^43^, oxidative hydrolysis of oxime^44^, oxidation of p-methoxyphenol and p-alkoxyaniline^45^, oxidative hydrolysis of hydrazides^46^, oxidation of diaminomaleonitrile-derived Schiff base^47^, electrophilic oxidation of N-heterocyclic carbene (NHC) boranes^48^, oxidation of a sulfur atom^49^, and oxidation of N-heterocyclic based on radical cation mechanism^27,28^. To explore the chemical reaction between sensors and ClO^−^, the reaction products were investigated using ^1^HNMR and mass spectroscopies, FTIR, and computational studies. At low ClO^−^ concentrations, the product of oxidation was a cationic radical, as shown in Fig.S9.

According to the published literature^50,51^, radical scavenging is employed by DMSO, t-butanol, and gallic acid to detect the radical cation mechanism. According to Fig.S10, scavenging could be seen by the naked eye and was also confirmed by the loss of the developed absorption peaks for IBTQ and DMP-BTQ at 588 nm and 580 nm, respectively. The absorbances for IBTQ and DMP-BTQ drop as scavenger concentrations increase. The antioxidant’s method for scavenging cyanine radicals was depicted in Fig. S11. This was supported by ^1^HNMR, mass spectrometry, and FTIR. For IBTQ, ^1^HNMR in DMSO-d_6_ solution (Fig. S12) shows a signal at 4.80 ppm (N-CH_2_) was present before ClO^−^ addition that is shifted to 4.84 ppm (N.^+^-CH_2_) after the addition of ClO^−^. For DMP-BTQ, ^1^HNMR in DMSO-d_6_ solution (Fig. S13) shows a signal at 4.78 ppm (N-CH_3_) that was present before ClO^−^ addition. This peak is shifted to 4.84 ppm (N.^+^-CH_3_) after ClO^−^ addition. Further study was carried out using mass spectrometry for IBTQ and DMP-BTQ in PBS. The reaction solution for IBTQ showed a signal at m/z = 574.19 that was assigned to the radical cation product maintaining the same molecular mass as the parent IBTQ (Fig. S15). The signal peak for DMP-BTQ in the presence of ClO^−^ occurs at m/z = 708.15 assigned to the radical cation product as shown in Fig. S16. That signal peak occurs at the same value as the parent DMP-BTQ of m/z = 708.45. The existence of (C=N) in the sensors before and after ClO^−^ addition was linked to signals in the FTIR data at 1634 cm^− 1^ and 1643 cm^− 1^ for IBTQ and DMP-BTQ, respectively (Fig. S17). All these data confirmed the reaction between ClO^−^ and investigated sensors occurring through a radical cation mechanism in agreement with literature where 2,2′-Azino-bis(3-ethylbenzothiazoline)-6-sulfonic acid-diammonium salt (ABTS), benzothiazole cyanine dyes, and promethazine hydrochloride sensors underwent the radical cation mechanism with hypochlorite^27,28,52^. The higher solubility of sensors radical cations compared with the parent cyanine sensors explains the QCM data. It is very useful if a sensor can be reversible in nature and be reusable for selectively sensing hypochlorite. To test the reversibility and reusability of sensors, the absorption spectral changes of sensors with increasing addition of antioxidants such as DMSO, t-butanol, and gallic acid are shown in Fig. S10. There is a gradual decrease at about 580 nm, while a new band at about 505 nm appears. The UV-vis spectra can almost return to the free sensor state of IBTQ and DMP-BTQ after the addition of antioxidants.

The reversible and reusable responses of the sensors were demonstrated by carrying out four alternate cycles of the titration of sensors with ClO^−^ followed by the addition of Gallic acid (Fig. S18). Titration of sensors with ClO^−^ ion results in remarkably increasing the values of A_588nm_/A_505nm_ ratios for IBTQ and A_580nm_/A_509nm_ for DMP-BTQ due to the formation of radical cation product. Titration of the radical cation with Gallic acid results in the scavenging of radical cation and return to free sensors, which is accompanied by a significant increase in absorbance at 505 nm and 509 nm for IBTQ and DMP-BTQ, respectively. The repeated ON/OFF behavior of UV-vis as well as color changes under ambient light from red to blue and then back to red (Inset Fig. S18) demonstrates the reversibility and reusability of the studied sensors.

### Computational investigations

#### Optimized molecular structures (MSs)

Cyanine sensors MSs were optimized in ethanol using the DFT/M06-2X/LANL2DZ method. The optimized MSs’ results are shown in Fig. S19. For IBTQ and DMP-BTQ optimized MSs, certain significant selected optimized MS parameters have been examined, including bond length in Å, bond angle (°), and dihedral angle (°). The results are compiled in Table S4. Among the significant deductions are the following: (i) According to the published dihedral angle values, the examined MSs are not planar as the quinoline rings in IBTQ and DMP-BTQ rotate away from the benzothiazole via 42.08° and 40.62°, respectively, to prevent steric hindrance. (ii) The overall hybridization type for the MSs under consideration is sp^2^ based on the provided bond angle values. (iii) The obtained bond lengths fluctuate because all the C-C, ring’s C-N bonds are either doubly bound or partially multiple bonded. (iv) The positive absorption frequencies in the IR spectra seen in Fig. S19 imply that the structures created are geometrically stable.

#### HOMO/LUMO MOs

Notably, the explanation of the electronic characteristics of the systems under discussion depends on the description of the HOMO/LUMO (H/L) molecular orbitals and their energy gaps^53^. H energy (E_H_) is used to characterize its ability to donate electrons, whereas L energy (E_L_) is used to describe its ability to extract electrons^54^. Therefore, in IBTQ, benzothiazole acts as an acceptor and quinoline as a donor; however, in DMP-BTQ, the roles are reversed, as illustrated in Fig. S20. The energy gap (ΔE) is represented as the difference between E_H_ and E_L_^54^. As shown in Fig. S20 H, H-1, L, and L + 1 MOs in IBTQ are localized over benzothiazole and quinoline groups, whereas H-2 and L + 2 MOs are localized over iodide atoms and other sub atoms. The graphical depiction of H/L, H-1/L + 1, and H-2/L + 2 for IBTQ and DMP-BTQ is shown in Fig. S20. Furthermore, as illustrated in Fig. S20, DMP-BTQ’s H, H-1, H-2, L, L + 1, and L + 2 MOs are localized on diverse sub-atoms. The energy levels of H, H-1, H-2, L, L + 1, and L + 2 as well as the ΔE values in ethanol solvent are computed. The computed E_H_ and E_L_ values are (− 6.757 and − 6.417 eV) for IBTQ and (-1.988 and − 2.074 eV) for DMP-BTQ. Comparing the values of E_H_, E_H-1_, E_H-2_, E_L_, E_L+1_, and E_L+2_, IBTQ has greater H, H-1, and H-2 stability than DMP-BTQ. The L, L + 1, and L + 2 of DMP-BTQ, on the other hand, are more stable than IBTQ. All studied MSs’ ΔE_1_ values are provided in the following order: IBTQ is higher than DMP-BTQ. These results show that DMP-BTQ is less stable than IBTQ. Fig. S19 makes it abundantly evident that the chemical makeup of organic substances has a significant impact on the energy gap. While DMP-BTQ has a smaller energy gap (ΔE_1_ = 4.342, ΔE_2_ = 5.849, and ΔE_3_ = 6.242 eV), IBTQ is distinguished by a high energy gap (ΔE_1_ = 4.769, ΔE_2_ = 7.890, and ΔE_3_ = 8.216 eV).

According to Fig. S20, the difference between the values of ΔE for IBTQ and DMP-BTQ indicates that the absorption spectrum of DMP-BTQ shifts to a longer wavelength because DMP-BTQ has a higher conjugation than IBTQ, which is consistent with the results of the experiment.

#### Quantum stability chemical (QSC) parameters

Utilizing E_L_ and E_H_ values, significant quantum stability chemical (QSC) characteristics including dipole moment (µ), electronegativity (χ), chemical potential (ρ), binding energy (BE), and chemical hardness (η) per unit atom were determined^55^. These QSC parameters are calculated using the equations: $\:\varvec{\uprho\:}=\frac{{E}_{H}+\:{E}_{L}}{2}\:$, **χ**$\:=\:-\frac{{E}_{H}+{E}_{L}}{2},\:\text{a}\text{n}\text{d}\:\varvec{\upeta\:}=\frac{{E}_{L}-{E}_{H}}{2}$^56^. Noteworthy, when a structure has a high µ; the electrical charge distribution is asymmetric. As shown in Table S5, DMP-BTQ has the greatest µ value compared to IBTQ. As a result, intramolecular charge transfer is more active in DMP-BTQ than in IBTQ. For ρ value, IBTQ has a lower value than DMP-BTQ, as seen in Table S5. This data shows that the escaping electrons from IBTQ are lower than those from DMP-BTQ. IBTQ, on the other hand, has the largest value of **χ** and consequently the greatest potential to attract electrons when compared to DMP-BTQ. Furthermore, IBTQ has a higher η value than DMP-BTQ. Those results indicate that IBTQ is undeniably challenging to free electrons, while DMP-BTQ possesses an extraordinary chance to offer electrons to another acceptor compounds.

#### Absorption spectra and molecular electrostatic potentials (MEP)

The electrophilic vs. nucleophilic sites of IBTQ and DMP-BTQ are demonstrated by Molecular Electrostatic Potential (MEP) assays, which are useful for detecting reaction sites^57^. The MEPs can be shown in Fig. S21. As illustrated in Fig. S21 (a and b), the neutral, negative, and positive positions at MEP interfaces are denoted by green, red, and blue colors. In the compounds under discussion, a negative region (red color) around the F, I, and N atoms shows that these sites are nucleophilic. The H atom and the C linkages to the N and S atoms were revealed in the positive (blue color) zones of IBTQ and DMP-BTQ, indicating that these sites are vulnerable to electrophilic attack.

According to Table S6, the experimental UV-Vis. absorption spectra of these particles demonstrate that their practical maximum absorption wavelengths are 505 and 509 nm for IBTQ and DMP-BTQ, respectively (see Fig. 2). The π- π* electronic transition is the cause of the experimental electronic absorption shown in Fig. 2. Using the TD/M06-2X/LANL2DZ approach, the computational electronic absorption spectra for IBTQ and DMP-BTQ were determined in this study^56^. The outcomes are displayed in Fig. S21c, with Table S6 containing the relevant statistics. According to Table S6, the estimated electronic absorption spectra of IBTQ show three electronic transitions at absorption wavelengths of 442.12, 292.92, and 282.91 nm, respectively. The electronic transitions H→L, H-1→L, and H→L + 1 correspondingly are the cause of this absorption. Thus, the wavelength at which the experimental data match the most is 442.12 nm due to H→L. Using the same technique, Table S6 shows that DMP-BTQ has three electronic transitions at absorption wavelengths 469.52, 446.39, and 438.46 nm. Due to H→L, the absorption wavelength of 469.52 nm agrees with the observed spectrum.

#### NBO analysis

The NBO approach provides a useful foundation for researching charge transfer or conjugative interactions in molecular systems, as well as an efficient method for investigating inter- and intramolecular bonding^57^. NBO analysis at the M06-2X/LANL2DZ level of theory is used to calculate the second-order perturbation energies (stabilization or interaction energies) (E^2^ in kcal/mol) and the most significant interaction between Lewis’s type NBOs (donor) and non-Lewis NBOs (acceptor) for all studied MSs. The gathered data are summarized in Table S7. According to the NBO analysis results for IBTQ and DMP-BTQ MSs, a strong hyper conjugative interaction exists. The stabilized energy values are listed in Table S7. The delocalization of the π-bond from the C1-C6 to the C2-C3, C2-C3 to the C4-C5, C14-C16 to the C19-C22, and finally the C19-C22 to the C18-N29 stabilizes IBTQ by losses of 30.31, 30.87, 28.06, and 45.34 kcal/mol. Also, for DMP-BTQ, the delocalization of π bonds from the C1-C2 to the C3-C4, C2-C3 to the C4-C5, from the C14-C16 to C19-C22, and finally from the C19-C22 to the C18-N29 stabilize the DMP-BTQ by losses 23.98, 34.52, 28.02 and 45.70 kcal/mol.

### Paper strips for the detection of hypochlorite ions

Finally, to enable quick and easy detection, we prepared portable test paper strips for detecting ClO^-^ at low concentrations at the ppm level^58^. After applying the sensor solution (1 mM) to a paper strip, it was allowed to air dry. The paper strip changed from red to violet after being dipped in the ClO^-^ solution at 80 ppm and 12 ppm concentrations for IBTQ and DMP-BTQ, respectively (Fig. S23).

### The cytotoxicity of sensors in living cells

To determine the cytotoxicity effects of sensors on normal PBMCs, we treated cells in vitro with two sensors and evaluated their cytotoxic effects. We found that treatment of cells with IBTQ (high conc 100%) induced decreased significantly in the viable cells rather than medium conc (50%) and low conc (25%) by 2.8- fold as compared to control p-value ≤ 0.001, assessed by trypan blue exclusion assay. However, there was no significant change in the total number of viable cells after treatment with DMP-BTQ with different concentrations as compared to the control p-value = 0.04 as shown in Fig S24.

## Conclusions

In conclusion, we synthesized new sensors, IBTQ and DMP-BTQ, that can be efficiently developed as colorimetric sensors and QCM probes for detecting ClO^−^ ions with a high degree of sensitivity and selectivity. Because the LOD and LOQ values are in the ppm range, ClO^−^ detection is possible in physiological circumstances. The QCM approach revealed a linear relationship between frequency variations and ClO^−^ concentrations with a low limit of detection and quantification. Other advantages of the reported cyanine sensors include their simplicity, quick reaction time, high sensitivity, and low cost. The sensing process is described in terms of ClO^−^ oxidizing activity, which results in the formation of colored cyanine radical cations. To create theoretical spectra and evaluate the lowering effect of cyanine sensors via electron loss, computational studies were carried out. Radical scavengers such as DMSO, gallic acid, and t-butanol promoted sensor recovery, demonstrating radical production. The hue shift caused by trace ClO^−^ activity may be seen with the naked eye, making it helpful in monitoring hypochlorite levels in tap and drinking water.

## Electronic supplementary material

Below is the link to the electronic supplementary material.

Supplementary Material 1

## Data Availability

“All data generated or analyzed during this study are included in this published article.”
