# Supplementary material for: Monocationic versus dicationic-based monomethine cyanine dyes for ultrasensitive colorimetric detection of hypochlorite ion in water
Source: Sci Rep. 2025 Feb 15;15:5566. doi: 10.1038/s41598-025-88839-y (PMC11830026; doi:10.1038/s41598-025-88839-y)
Supplement: Supplementary file 1 — Supplementary Material 1 [file 41598_2025_88839_MOESM1_ESM.docx]

**Supporting information for**

**Monocationic Versus Dicationic-based Monomethine Cyanine Dyes for Ultrasensitive Colorimetric Detection of Hypochlorite Ion in Water**

Nermeen S. Hafez ^a*^, Wael A. Amer^a,b^, Ehab A. Okba^a^, Mahmoud A. S. Sakr^c^, Hussein H. Alganzory^d^, Sohaila M. Khalil^e^ and El-Zeiny M. Ebeid^a^

^a^ Chemistry Department, Faculty of Science, Tanta University, Tanta 31527, Egypt.

^b^ Chemistry Department, College of Science, Bahrain University, Sakhir 32038, Bahrain.

^c^ Center of Basic Science, Misr University for Science and Technology, 6^TH^ of October City, Egypt.

^d^ Chemistry Department, Faculty of Science, Benha University, Benha, Egypt.

^e^ Immunology and Parasitology Division, Zoology Department, Faculty of Science, Center of Excellence in Cancer Research, New Tanta University Teaching Hospital, Tanta, University, Egypt.

^*^Corresponding author: Nermeen S. Hafez, [Nermin.Salah@science.tanta.edu.eg](mailto:Nermin.Salah@science.tanta.edu.eg)

|  | **Content** |
| --- | --- |
| **Experimental section** | Materials and instruments  Synthesis of cyanine sensors  UV-Vis. spectral measurements  Quartz crystal microbalance )QCM) measurements  Real water samples test  Computational Studies  Paper-strip preparation  Separation of peripheral blood mononuclear cells (PBMCs)  Cell culture and treatments |
| **Fig. S1** | Microwave synthesis of cyanine–derivatives used as IBTQ (a) and DMP-BTQ(b) |
| **Fig. S2** | **a)** Frequency response (∆*f*) of QCM cell-coated IBTQ film as a function of time at different ClO^-^ concentrations **b)** Linear relation between ∆*f* responses and ClO^-^ doses (0.42, 0.81, 1.18, 1.53) for IBTQ **c)** Frequency response (∆*f*) of QCM cell-coated DMP-BTQ film as a function of time at different ClO^-^ concentrations and **d)** Linear relation between ∆*f* responses and ClO^-^ doses ( 0.142, 0.28, 0.416, 0.55, 0.68) for DMP-BTQ. |
| **Fig. S3** | Time-dependent changes of the absorbances of **a**) IBTQ (15 µM) at 588 nm upon addition of 0, 3.57, 7.15, 18.59 ppm ClO^-^, **b**) DMP-BTQ (10 µM) at 580 nm upon addition of 0, 3.57, 8.58, 17.16 ppm ClO^-^ in ethanolic / PBS (10 mM, pH = 7.4). Effects of pH on the absorbances of **c**) IBTQ (15 µM) in the absence and presence of ClO^-^ (15 ppm) at 588nm; **d**) DMP-BTQ (10 µM) in the absence and presence of ClO^-^ (2 ppm) at 580 nm. |
| **Fig. S4** | Kinetics of **a**) IBTQ (15 µM); **b**) DMP-BTQ (10 µM) at 505 nm upon addition of 17.16 ppm ClO^-^ in ethanolic / PBS (10 mM, pH = 7.4). |
| **Fig. S5** | Time-dependent changes of the absorbances of IBTQ (15 µM) at 588 nm upon addition of 7.15 ppm ClO ^–^ and DMP-BTQ (10 µM) at 580 nm upon the addition of 8.58 ppm ClO^-^ in ethanolic / PBS (10 mM, pH = 7.4) after storage for different times (1 min, 10 min, 30min, 1h, 2h, 5h, 10h, 15h, 20h, 36h, 48h, 60h, 84h, 132h, 144h and 192 h) under ambient condition. |
| **Fig.S6** | Solvent effect on the absorbances of IBTQ (15 µM) at 505 nm and DMP-BTQ (10 µM) at 509 nm. |
| **Fig. S7** | The absorbances of **a)** IBTQ with ClO^-^ (10 ppm) at 588 nm; **b)** DMP-BTQ with ClO^-^ (15 ppm) at 580 nm versus sensor concentrations in ethanolic / PBS (10 mM, pH = 7.2). |
| **Fig. S8** | Linear relationship between absorbance of DPD (0.75 mM) as a function of ClO^-^ concentrations. |
| **Fig. S9** | **a)** The sensing reaction mechanism between IBTQ and ClO^-^. **b)** The sensing reaction mechanism between DMP-BTQ and ClO^-^. |
| **Fig. S10** | **a)** Absorption spectra of IBTQ (15 µM) and ClO^-^ (45 ppm) in the presence of 80µM of antioxidants. **b)** Correlations between absorbance and antioxidant concentrations at 588 nm. **c)** Absorption spectra of DMP-BTQ (10 µM) and ClO^-^ (5 ppm) in the presence of 100 µM of antioxidants. **d)** Correlations between absorbance and antioxidant concentrations at 580 nm. |
| **Fig. S11** | Gallic acid as an antioxidant scavenger for the studied cyanine radical cations. |
| **Fig. S12** | ^1^H NMR of a) IBTQ and b) IBTQ with ClO^-^ in DMSO-*d_6_*. |
| **Fig. S13** | ^1^H NMR of a) DMP-BTQ and b) DMP-BTQ with ClO^-^ in DMSO -*d_6_*. |
| **Fig. S14** | ^13^C NMR of a) IBTQ and b) DMP-BTQ in DMSO-*d_6_* |
| **Fig. S15** | EI-MS for a) IBTQ and b) IBTQ after the addition of ClO^-^. |
| **Fig. S16** | EI-MS for a) DMP-BTQ and b) DMP-BTQ after the addition of ClO^-^. |
| **Fig. S17** | FTIR for a) IBTQ before and after the addition of ClO^-^, b) DMP-BTQ before and after the addition of ClO^-^. |
| **Fig. S18** | Reusability graph of a) the relative absorbance ratio (A_588nm_/A_505nm_) of IBTQ (15 µM) during the addition of 7.15 ppm ClO ^–^ and gallic acid; b) The relative absorbance ratio (A_580nm_/A_509nm_) of DMP-BTQ (10 µM) during the addition of 8.58 ppm ClO^-^ and gallic acid in ethanolic / PBS (10 mM, pH = 7.4). Insets: Visual color changes after each sequential addition of ClO^-^ and gallic acid under ambient light. |
| **Fig. S19** | (**Top**) IBTQ and DMP-BTQ optimized with labeled front, side, and (**bottom**) IR spectra in ethanol using the M06-2X/LANL2DZ measurement theory. |
| **Fig. S20** | Graphic illustration of H/L, H-1/L+1, and H-2/L+2 energies, molecule distribution, and energy gaps (ΔE) for IBTQ and DMP-BTQ. |
| **Fig. S21** | M06-2X/LANL2DZ electrostatic potential map (ESP) level of theory for **a)** IBTQ and **b)** DMP-BTQ. **c)** The absorption spectra estimated for both IBTQ and DMP-BTQ using the TD/ M06-2X/LANL2DZ technique. |
| **Fig. S22** | IBTQ (15 µM) **(a)** and DMP-BTQ (10 µM) **(b)** color change upon varying ClO^-^ concentrations. |
| **Fig. S23** | Photographs of a test paper-strip for ClO^−^ detection using **a**) IBTQ with 0 and 30 ppm ClO^-^ and **b**) DMP-BTQ with 0 and 15 ppm ClO^-^. |
| **Fig. S24** | **PBMCs viability after treatment with two sensors**: Number of viable cells after treatment with IBTQ (A) DMP-BTQ (B). Cells were treated with (High (100%), Medium (50%), and low (25%) overnight, harvested washed, and then counted***p value ≤0.001 |
| **Table S1** | Experimental (based on UV-Vis. spectrophotometer signal) versus calculated LOD values of IBTQ and DMP-BTQ. |
| **Table S2** | Comparison of LOD values of IBTQ and DMP-BTQ with those of some previously reported HOCl/OCl^-^ probes in literature. |
| **Table S3** | Determination of ClO^-^ in real water samples using IBTQ and DMP-BTQ versus DPD using UV-Vis. absorption. |
| **Table S4** | Optimized structural parameters (bond length in Å, bond angle (^o^), and dihedral angle (^o^) computed for IBTQ and DMP-BTQ. Refer to Fig. S19 for labeling instructions. |
| **Table S5** | Quantum chemical characteristics for IBTQ and DMP-BTQ in ethanol, E_H_, E_L_, energy gap (E_g_), dipole moment (μ), electronegativity (χ), chemical potential (ρ), and chemical hardness (η). |
| **Table S6** | Calculated electronic absorption parameters for IBTQ and DMP-BTQ. |
| **Table S7** | Selection of most influential second-order perturbation (E^2^) estimation of the hyper conjugative energies (kcal/mol) of IBTQ and DMP-BTQ molecular modeling structures. |

**1. Experimental section**

**1.** **1 Materials and instruments**

As previously reported in the literature, IBTQ and DMP-BTQ were synthesized using a microwave-assisted method ^1,2^. All the chloride and potassium salts, calcium hypochlorite, hydrogen peroxide, and t-butanol used were obtained from Sigma-Aldrich. Ethanol (99.5%) was obtained from Chem-Lab.

UV-Vis. spectra were collected using a Shimadzu UV-50 UV-Vis spectrophotometer. The electronic absorption spectra were recorded by using matched quartz cuvettes with a 1 cm path length. All experiments were performed at room temperature. The ^1^H NMR spectra (400 MHz) and ^13^C NMR spectra (101 MHz) were acquired at 25 °C in DMSO-d6 utilizing tetramethylsilane as an internal standard on a JEOL. The elements analysis was conducted with a Perkin-Elmer 240 CHN elements analyzer. The pH was measured with an Adwa AD1020 Professional pH-ORP-TEMP Bench Meter (2.00~ 16.00 pH). Quartz crystal microbalance (QCM) measurements were made using an AT-5 MHz cut quartz crystal coated on each side with electrodes made of gold and a GW frequency counter (Model GFC-8055G) to calculate the resonance frequencies. FT-IR spectra were obtained in the 4000 to 400 cm^-1^ range using a JASCO FT/IR-4100 spectrophotometer. A Finnigan MAT 8222 EX mass spectrometer was used to obtain the EI-mass spectra at 70 eV. An XT-4 micromelting melting equipment was used to measure the melting points. A Start S Milestone S/N 129802 microwave oven was used for all the microwave experiments. The kinetic measurements were carried out with the Applied KinetAsyst SF-61DX2 stopped flow device (HI-Tech Scientific) and a Peltier thermostat.

**1. 2 Synthesis of cyanine sensors**

Cyanine sensors were prepared by microwave-assisted synthesis. For IBTQ, equivalent amounts of 3-ethyl-2-(methylthio)-3a,7a-dihydrobenzo[d]thiazol-3-ium tetrafluoroborate 0.906 gm (2 mmol) and 1-(4-iodobutyl)-4-methylquinolin-1-ium iodide 0.594 gm (2 mmol) were mixed in a glass conical flask. A few drops of trimethylamine (1 mL) were also used during this step as a catalyst. The mixture was subjected to microwave irradiation for 6 min at a power of 280 W. After cooling and washing with diethyl ether, an orange to yellowish-orange precipitate was obtained (Fig. S1a).

Synthesis of DMP-BTQ was performed in two steps. In the first one, a monomethine cyanine dye (1-(3-iodopropyl)-4-((3-methylbenzothiazol-2(3H)-ylidene)methyl)quinolin-1-ium) was prepared according to literature ^1^. In the second step, equivalent amounts of monomethine cyanine dye, the product from the first step, 0.586 gm (1 mmol), and N,N-dimethylpyridin-4-amine 0.122 gm (1 mmol) were stirred in the presence of DMF (20 mL). A little catalytic amount of trimethylamine (1 mL) was also used during this step. The mixture was subjected to microwave irradiation with stirring for 90 min and 100 W power. The formed precipitate was filtered off, washed with CH₂Cl₂, and dried at 60 ºC (Fig. S1b). The structures of synthesized sensors were characterized using ^1^H NMR (Fig. S12 and S13), ^13^C NMR spectra (Fig. S14), EI-mass (Fig. S15 and S16), FT-IR spectra (Fig. S17), and elemental analyses.

The physical and spectral criteria of -((3ethylbenzothiazol-2(3H)-ylidene)methyl)-1-(4-iodobutyl)quinolin-1-ium tetrafluoroborate (IBTQ) are: Yield 87%, Orange fine crystals, m.p. 218-221^o^C; (EI) m/z: calcd for [C_23_ H_24_ BF_4_N_2_ S I] 574.07; found, 574.19; IR(KBr): ν = 1473 (SH), 1639, 1229 cm^-1^ (C=C, C=N); ^1^HNMR (DMSO-d_6_): δ = 1.40 (t, 3H, CH_3_), 1.92 (m, 4H, 2CH_2_), 3.33 (t, 3H, ICH_2_), 4.66 (m, 4H, 2NCH_2_), 6.93 (s, 1H, =CH); 7.37-8.81 (m, 10H, Ar-H); ^13^CNMR: δ = 7.68, 26.38, 29.89, 53.02, 75.43 (5CH_2_), 12.33 (CH_3_), 87.53 (=CH), 108.01, 112.87, 118.14, 123.06, 124.12, 124.28, 124.64, 125.92, 126.89, 128.35, 133.3, 137, 139.59, 144.28, 148.82 (Ar-C), 159.30 (NCS); C_23_ H_24_ BF_4_N_2_ S I (574); Calcd: C, 48.11; H, 4.21, N, 4.88; S, 5.58; found: C, 48.1; H, 4.31; N, 4.87; S, 5.58.

The physical and spectral criteria of 1-(3-(4-(dimethylamino)pyridin-1-ium-1-yl)propyl)-4-((3-methylbenzothiazol-2(3H)-ylidene)methyl)quinolin-1-ium diiodide (DMP-BTQ) are: Yield 91%, reddish orange crystals, m.p.: 286-288 ^o^C; (EI) m/z: calcd for [C_28_H_30_I_2_N_4_S] 708.4; found, 708.45; IR(KBr): ν = 1473 (SH), 1643, 1266 cm^-1^ (C=C, C=N); ^1^HNMR (DMSO-d_6_): δ (ppm) = 2.45 (m, 2H, CH_2_), 3.17 (s, 6H, 2CH_3_), 4.04 (s, 3H, CH_3_), 4.41 (t, J=7.0 Hz, 2H, CH_2_), 4.69 (t, J=6.8 Hz, CH_2_), 6.92 (s, 1H, =CH), 7.02-8.81 (m, 14H, Ar-H); ^13^CNMR: δ (ppm) = 29.63, 51.39, 54.31 (3CH_2_), 34 (CH_3_), 39.7 (2CH_3_), 88.09 (=CH), 107.6, 108.03, 113.25, 118.08, 122.99, 124.74, 125.94, 126.69, 128.35, 133.37, 137.04, 140.3, 140.46, 144.31(Ar-C), 160.41 (NCS); C_28_H_30_I_2_N_4_S (708.4); Calcd: C, 47.47; H, 4.27, N, 7.91; S, 4.53; found: C, 47.27; H, 4.41; N, 8.09; S, 4.52.

**1. 3** **UV-Vis. spectral measurements**

Both sensors were dissolved in ethanol as a stock solution (0.1 mM) and then diluted to the concentration required for ClO^-^ detection. The ClO^-^ stock solution was prepared with deionized water and ClO^-^ ions as the raw material. Iodometry was used to confirm the content of ClO^-^ ions before usage. A phosphate-buffered saline was used to change the solution pH from 2 to 12. pH sensing experiments have been performed in high ionic strength buffer (PBS 10 mM, ionic strength ≃ 162.7 mM. To investigate ClO^-^ selectivity, stock solutions of cyanine sensors (0.1 mM) in ethanol and test ions (1 mM each) in deionized water were prepared.

**1.4** **Quartz crystal microbalance )QCM) measurements**

Gold electrodes were used with an AT 5 MHz quartz crystal for the QCM measurements. The crystal frequency was measured using a GW frequency counter. Additionally, the specifics of the QCM setting have already been described elsewhere ^3,4^. The resonance frequency was first recorded after washing and letting the gold electrode of the QCM electrode dry. Using a Hamilton micro syringe, 200 µl of the cyanine sensor's ethanolic solutions were injected onto the QCM gold electrode to form a cyanine sensor-coated QCM probe, which was then dried at 45°C. 100 ml of deionized water (DI water) was poured into the QCM's chamber. After establishing a stable baseline, the resulting frequency was recorded. The frequency stability indicates that the cyanine probe film adhered successfully to the QCM electrode. The frequency was then recorded when an exact concentration of calcium hypochlorite solution was injected into the probe chamber. Hypochlorite injection leads to soluble radical cation formation which undergoes leaching with subsequent mass loss and increased frequency. According to the Sauerbrey equation, the cyanine probe mass deposited onto the gold electrode of the QCM is related to the frequency change by the relation ^4^:

$\Delta f= -(2{f_{o}^{2}}_{.}/\sqrt{\rho_{Q}\mu_{Q}} ) m$ (Eqn. 1)

Where *f*_o_ (Hz) is the fundamental frequency of the quartz crystal,$\rho_{Q}$is the quartz density (2.649 g cm^-3^), and $\mu_{Q}$ is to the quartz shear modulus (2.947 ×10^11^ dyne cm^-2^).

**1. 5 Real water samples test**

Samples of tap water were gathered from various locations, including the Kafr El Zayat (KZ) and Tanta Water Treatment Stations at Gharbia Governorate, as well as distilled water and Aquafina bottled water from PepsiCo, without any prior treatment. After being exposed to hypochlorite ions, the absorption spectra of the cyanine sensors were examined.

**1.6 Computational Studies**

To study the optical and electronic properties of cyanine sensors, Density Functional Theory (DFT) simulations were performed using the Gaussian 16 program ^5^. The M06-2X ^6,7^, the LANL2DZ, and hybrid functional ^8,9^ basis set since they provide accurate results. The optical characteristics of IBTQ and DMP-BTQ were examined by performing time-dependent DFT computations for the first twenty excited states using the M06-2X/LANL2DZ level of theory. Additionally, the DFT/M06-2X/LANL2DZ method and the Natural Bond Orbital (NBO) analysis ^9,10^ were used to examine the properties of IBTQ and DMP-BTQ in ethanol.

**1.7 Paper-strip preparation**

Paper strips were made using readily available neutral filter papers. The filter paper was loaded with the cyanine sensor's ethanolic solution (0.1 mM) and allowed to air dry at room temperature. The filter sheets were then treated with direct drips of ClO^-^aqueous solutions.

**1.8 Separation of peripheral blood mononuclear cells (PBMCs)**

Blood samples (5 ml) were collected from volunteer; Samples were collected in EDTA tubes (BD Vacutainer, USA). Peripheral blood mononuclear cells (PBMCs) were separated by Ficoll-Hypaque density separation medium (Sigma Aldrich, USA), then washed twice by PBS.

**1.9 Cell culture and treatments**

PBMCs (700×10^3^ cells/ml) were seeded in 12 well plate in RPMI 1640 (Gibco, Life Technologies, Grand Island, NY, USA) containing 10% fetal bovine serum (FBS; Invitrogen, Carlsbad, CA, USA), 2 mmol/L L-glutamine 100 units/ mL of penicillin, 100 μg/ml of streptomycin. Cells were treated with two dyes and maintained at 37˚C in a humidified 5% CO_2_ atmosphere overnight with three different concentrations: High (100%), Medium (50%), and low (25%) for each sensor. The cells were harvested and then counted.

**1.10 Cell viability**

The effects of sensors on the cell viability were assessed by trypan blue exclusion assay. Cell lines were cultured and treated with sensors in different concentrations overnight as mentioned previously. Cells were then harvested and washed by PBS, and 0.2% trypan blue solution was added for 1 min then, cells were counted using a hemocytometer.

**
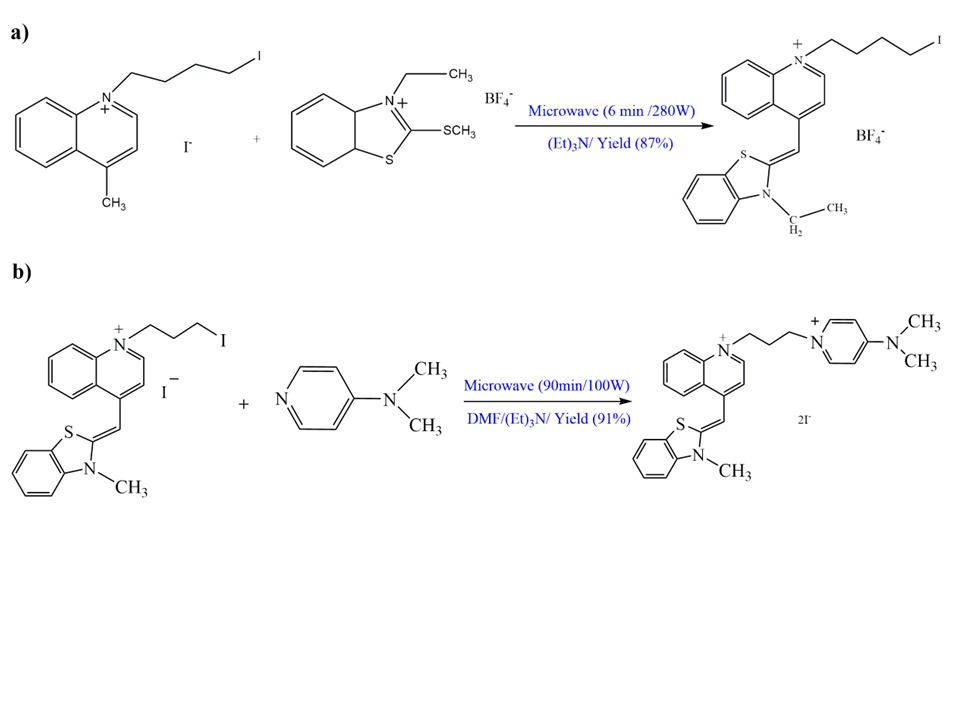
**

**Fig. S1** Microwave synthesis of cyanine–derivatives used as IBTQ (a) and DMP-BTQ(b)


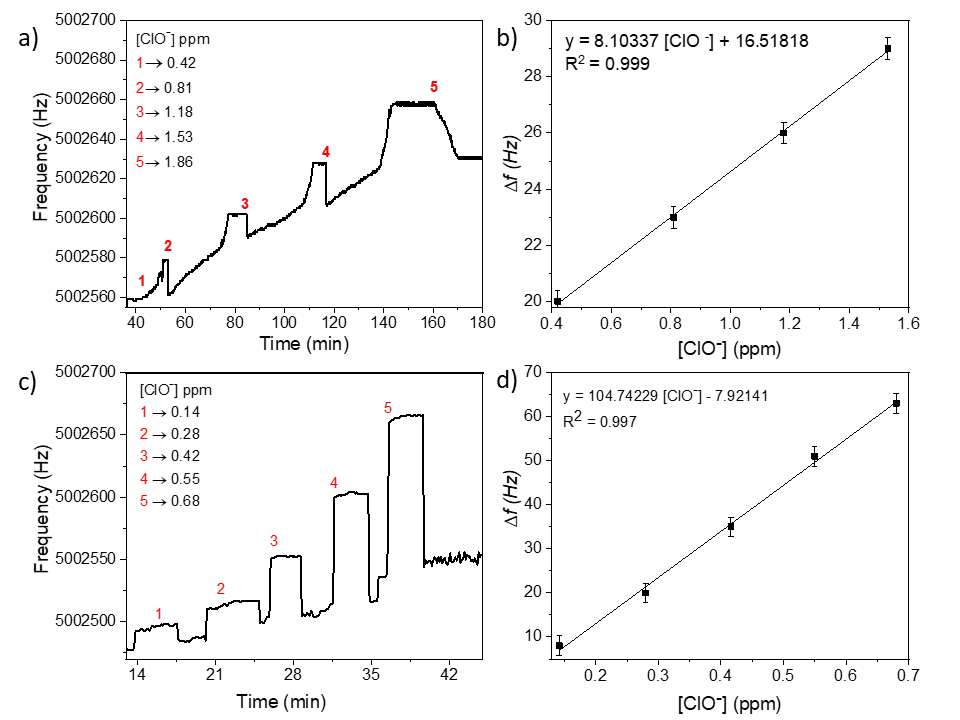


**Fig.S2 a)** Frequency response (∆*f*) of QCM cell-coated IBTQ film as a function of time at different ClO^-^ concentrations **b)** Linear relation between ∆*f* responses and ClO^-^ doses (0.42, 0.81, 1.18, 1.53) for IBTQ **c)** Frequency response (∆*f*) of QCM cell-coated DMP-BTQ film as a function of time at different ClO^-^ concentrations and **d)** Linear relation between ∆*f* responses and ClO^-^ doses ( 0.142, 0.28, 0.416, 0.55, 0.68) for DMP-BTQ.


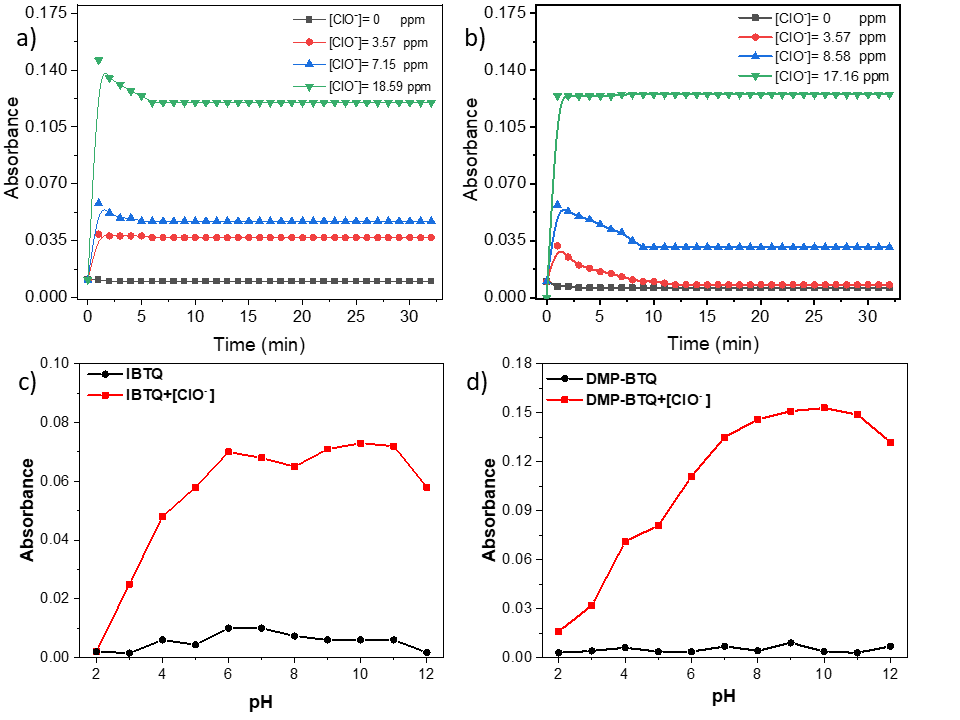


**Fig. S3** Time-dependent changes of the absorbances of **a**) IBTQ (15 µM) at 588 nm upon addition of 0, 3.57, 7.15, 18.59 ppm ClO^-^, **b**) DMP-BTQ (10 µM) at 580 nm upon addition of 0, 3.57, 8.58, 17.16 ppm ClO^-^ in ethanolic / PBS (10 mM, pH = 7.4). Effects of pH on the absorbances of **c**) IBTQ (15 µM) in the absence and presence of ClO^-^ (15 ppm) at 588nm; **d**) DMP-BTQ (10 µM) in the absence and presence of ClO^-^ (2 ppm) at 580 nm.


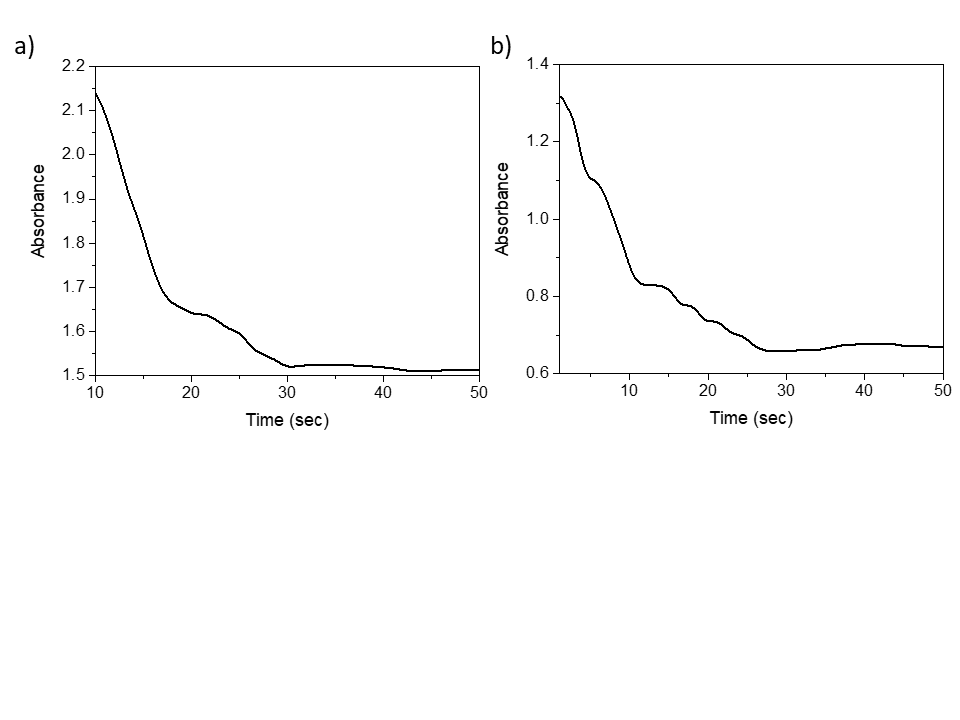


**Fig. S4** Kinetics of **a**) IBTQ (15 µM); **b**) DMP-BTQ (10 µM) at 505 nm upon addition of 17.16 ppm ClO^-^ in ethanolic / PBS (10 mM, pH = 7.4).


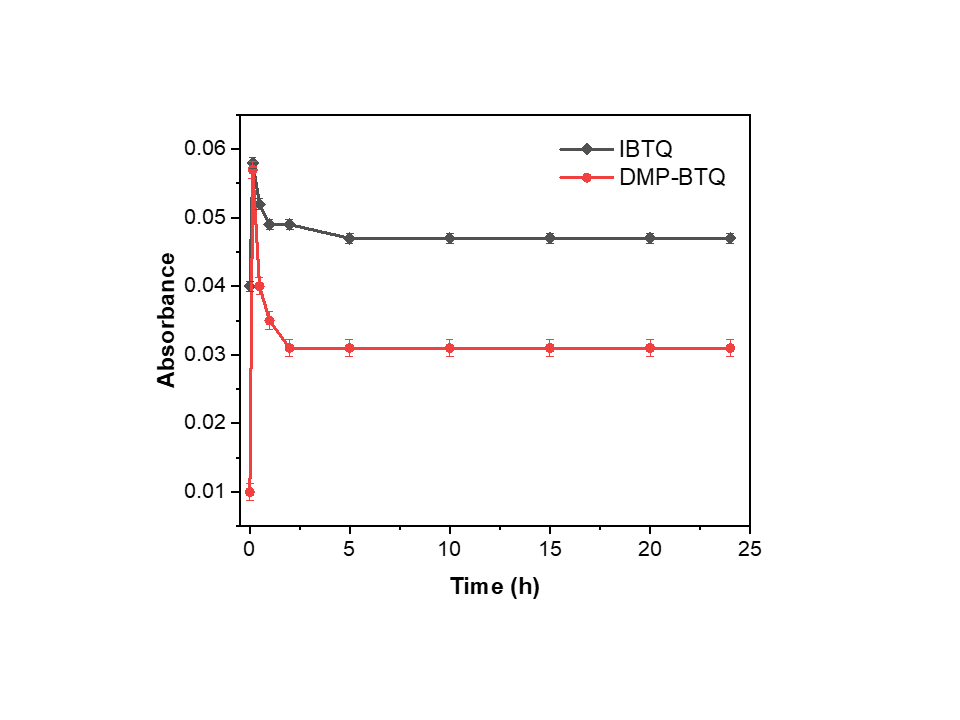


**Fig. S5** Time-dependent changes of the absorbances of IBTQ (15 µM) at 588 nm upon addition of 7.15 ppm ClO ^–^ and DMP-BTQ (10 µM) at 580 nm upon the addition of 8.58 ppm ClO^-^ in ethanolic / PBS (10 mM, pH = 7.4) after storage for different times (1 min, 10 min, 30min, 1h, 2h, 5h, 10h, 15h, 20h, 36h, 48h, 60h, 84h, 132h, 144h and 192 h) under ambient condition.


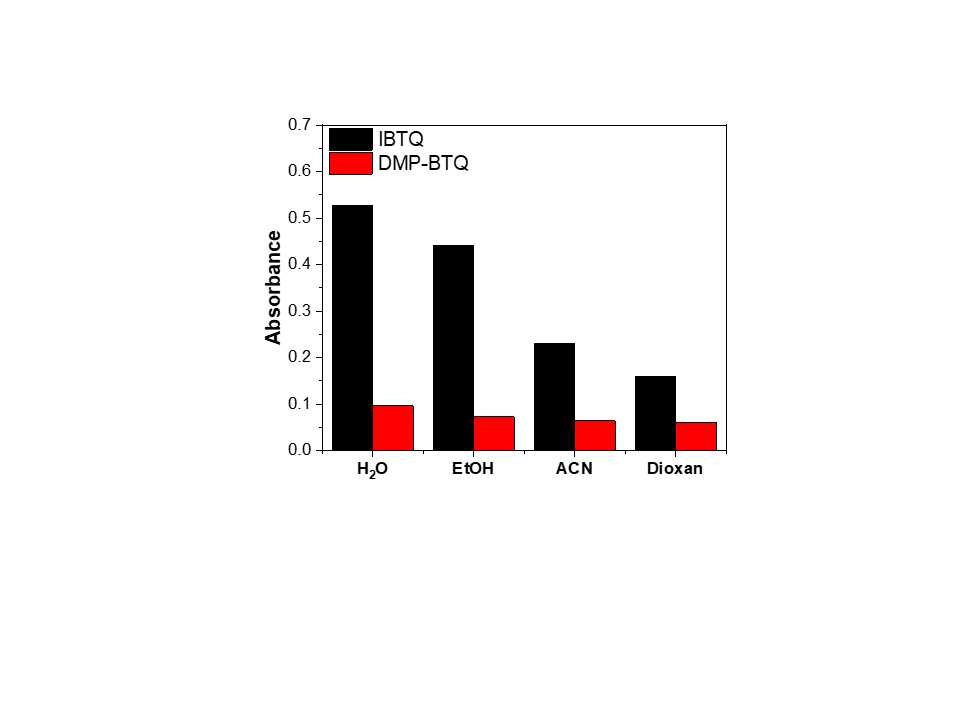


**Fig.S6** Solvent effect on the absorbances of IBTQ (15 µM) at 505 nm and DMP-BTQ (10 µM) at 509 nm.


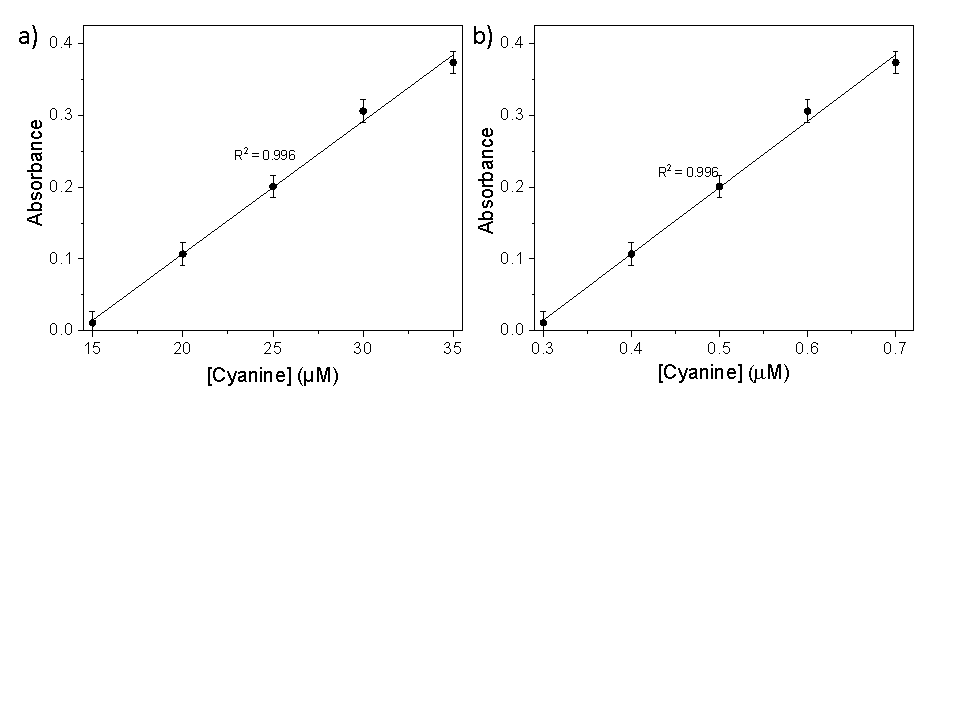


**Fig. S7** The absorbances of **a)** IBTQ with ClO^-^ (10 ppm) at 588 nm; **b)** DMP-BTQ with ClO^-^ (15 ppm) at 580 nm versus sensor concentrations in ethanolic / PBS (10 mM, pH = 7.2).


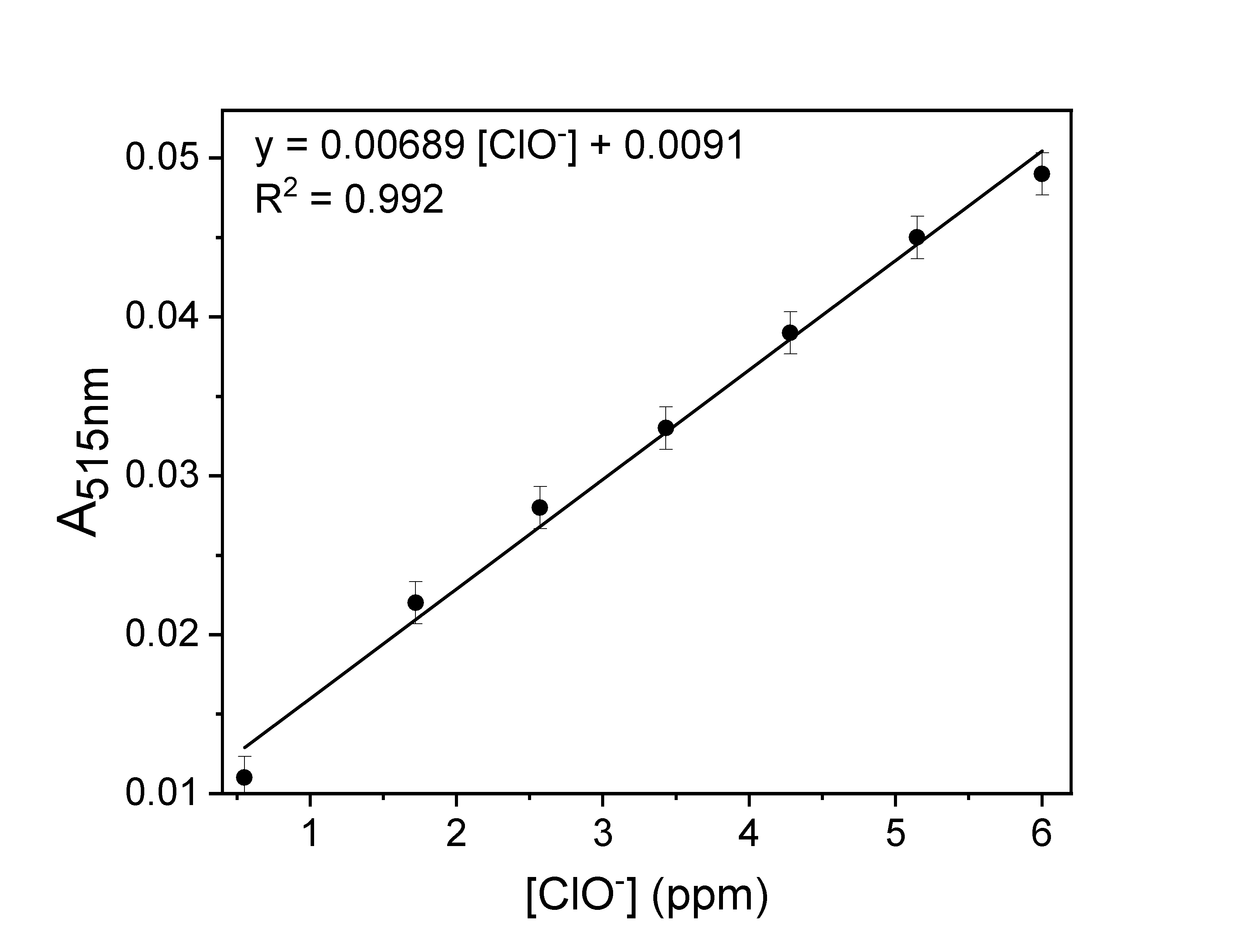


**Fig. S8** Linear relationship between absorbance of DPD (0.75 mM) as a function of ClO^-^ concentrations.


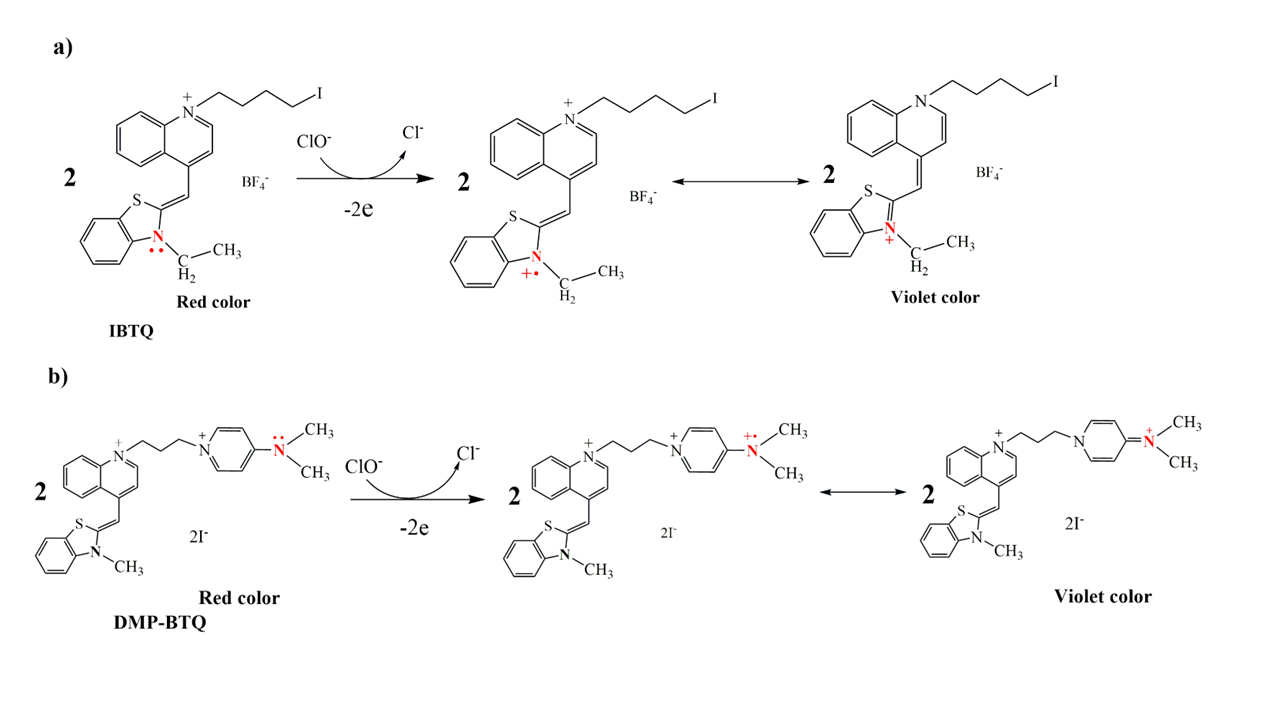


**Fig S9 a)** The sensing reaction mechanism between IBTQ and ClO^-^. **b)** The sensing reaction mechanism between DMP-BTQ and ClO^-^.

**
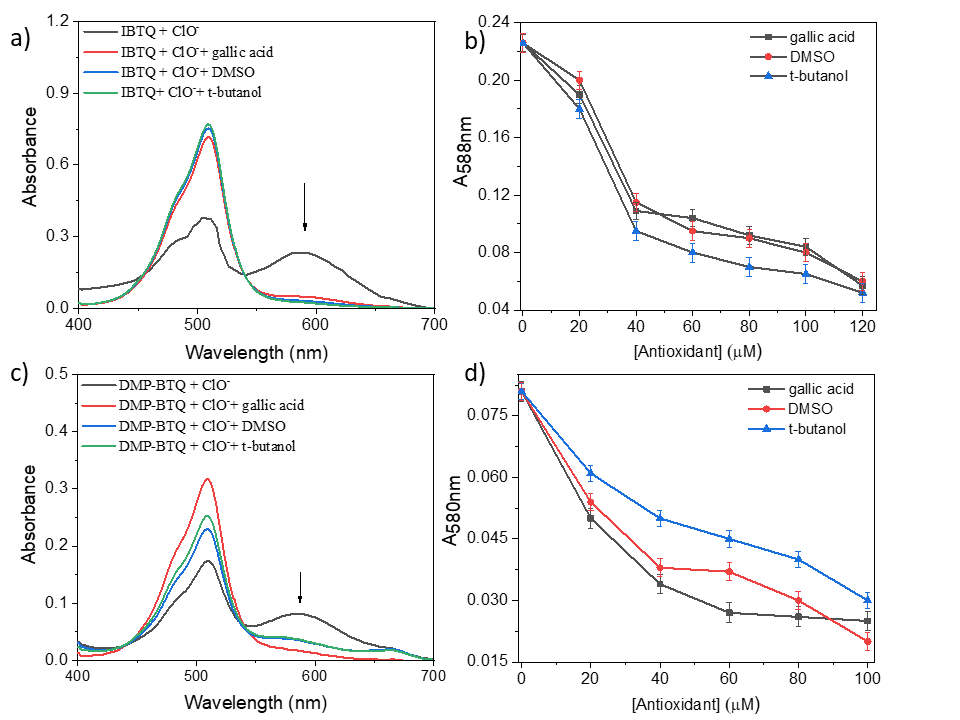
**

**Fig. S10** **a)** Absorption spectra of IBTQ (15 µM) and ClO^-^ (45 ppm) in the presence of 80µM of antioxidants. **b)** Correlations between absorbance and antioxidant concentrations at 588 nm. **c)** Absorption spectra of DMP-BTQ (10 µM) and ClO^-^ (5 ppm) in the presence of 100 µM of antioxidants. **d)** Correlations between absorbance and antioxidant concentrations at 580 nm.


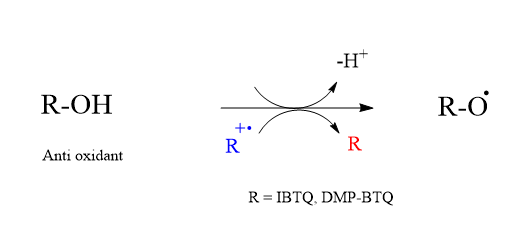


**Fig. S11** Gallic acid as an antioxidant scavenger for the studied cyanine radical cations.

| **a)** | 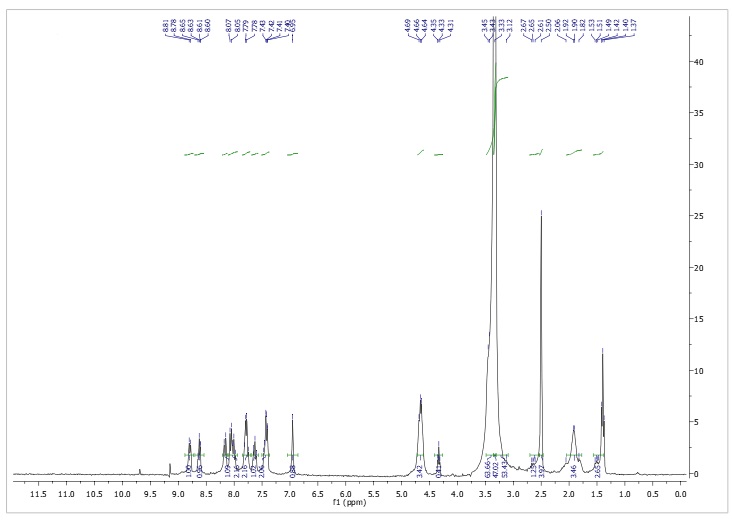 | |
| --- | --- | --- |
| **b)** | |  |

**Fig. S12** ^1^H NMR of a) IBTQ and b) IBTQ with ClO^-^ in DMSO-*d_6_*.

| **a)** | 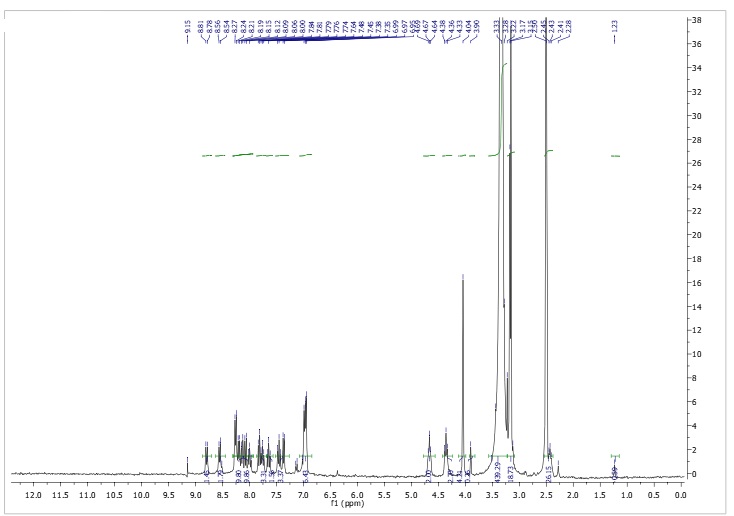 |
| --- | --- |
| **b)** |  |

**Fig. S13** ^1^H NMR of a) DMP-BTQ and b) DMP-BTQ with ClO^-^ in DMSO -*d_6_*.

| **a)** | **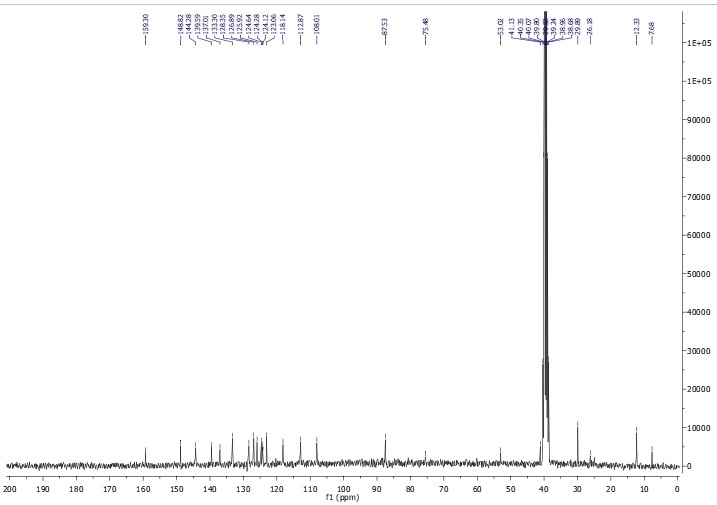** |
| --- | --- |
| **b)** | **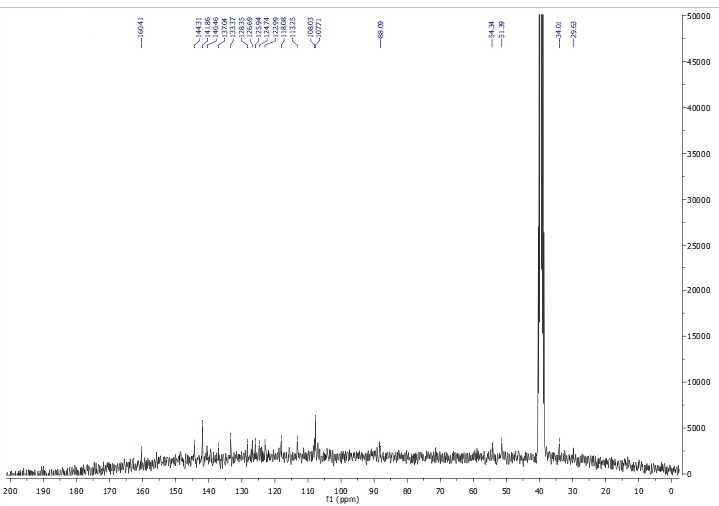** |

**Fig. S14** ^13^C NMR of a) IBTQ and b) DMP-BTQ in DMSO-*d_6_*.


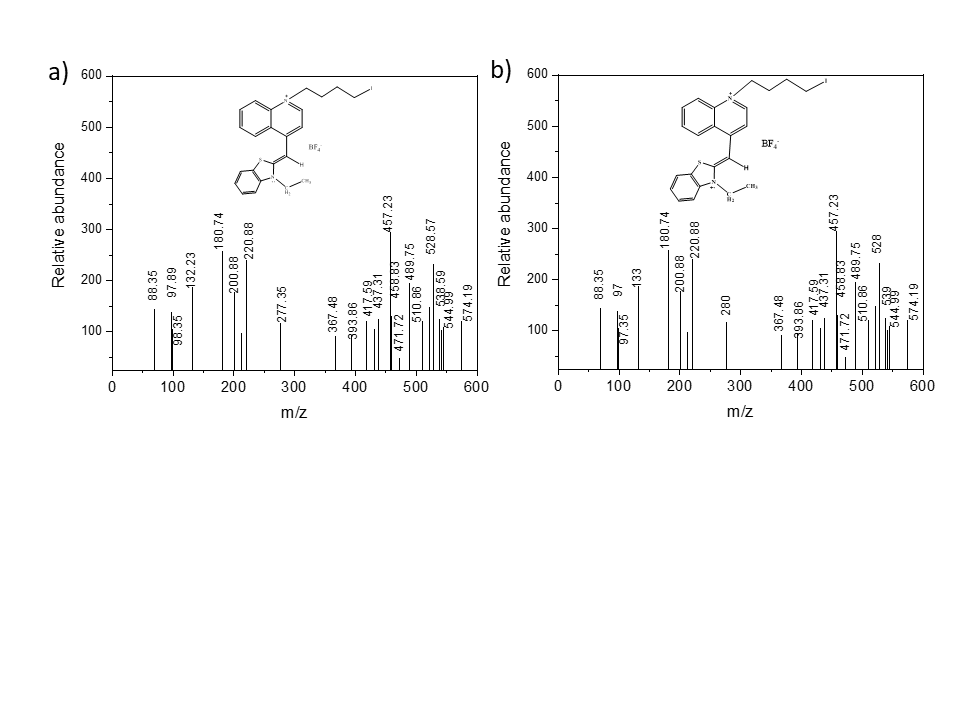


**Fig. S15** EI-MS for a) IBTQ and b) IBTQ after the addition of ClO^-^.

**
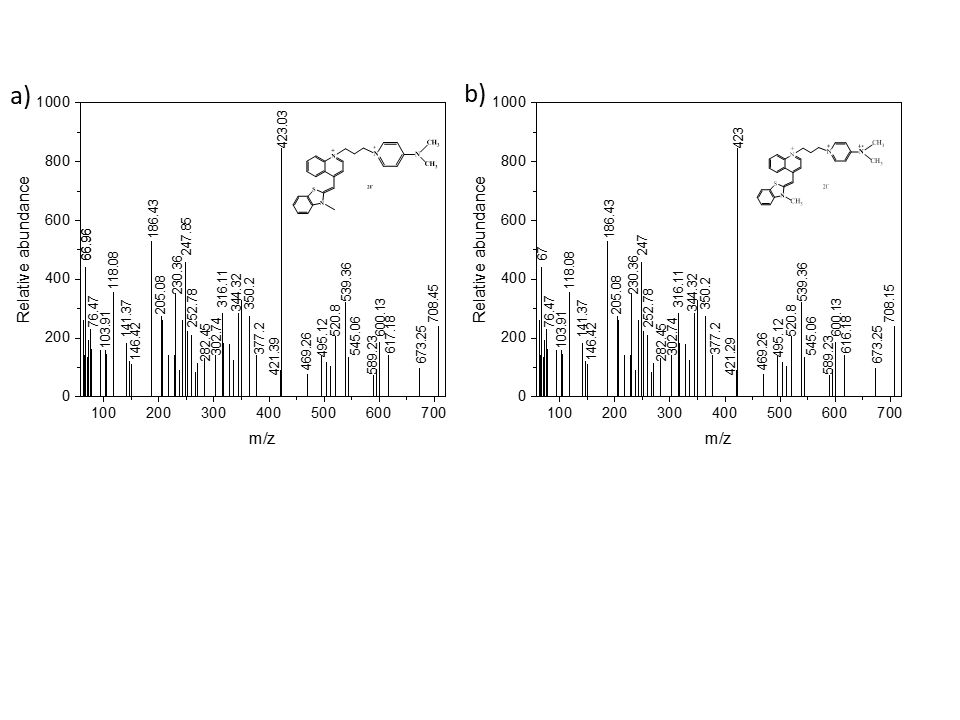
**

**Fig. S16** EI-MS for a) DMP-BTQ and b) DMP-BTQ after the addition of ClO^-^.


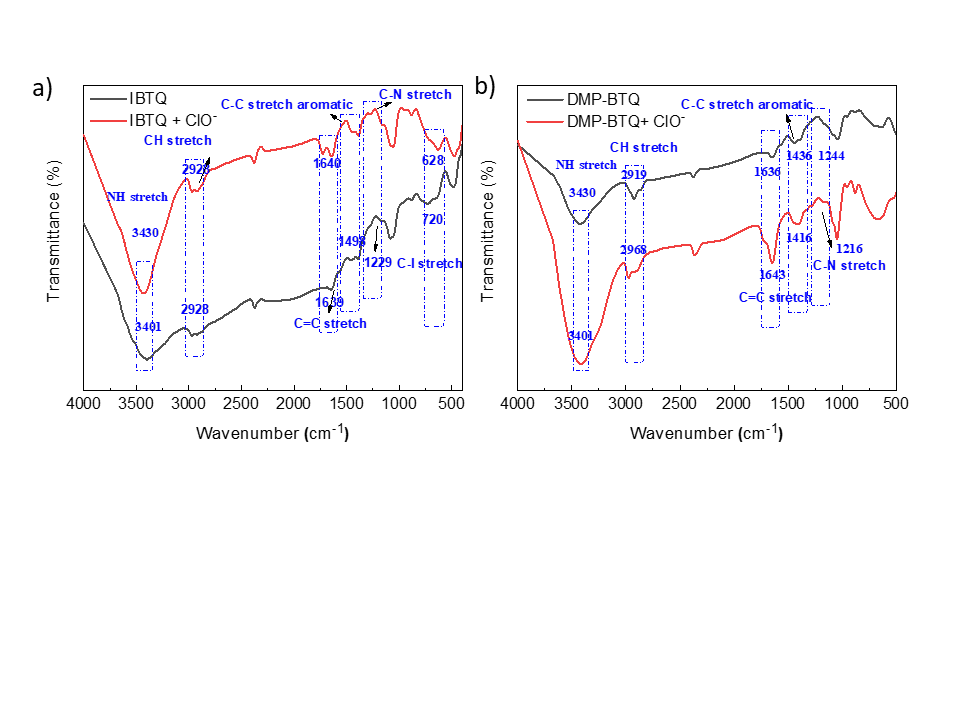


**Fig. S17** FTIR for a) IBTQ before and after the addition of ClO^-^, b) DMP-BTQ before and after the addition of ClO^-^.


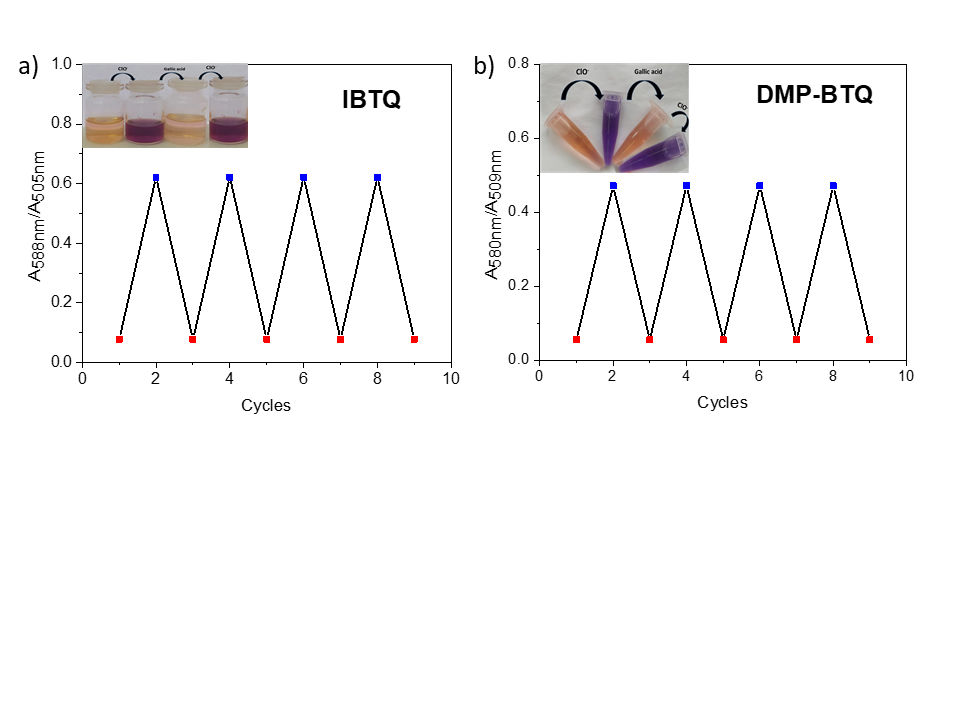


**Fig. S18** Reusability graph of a) the relative absorbance ratio (A_588nm_/A_505nm_) of IBTQ (15 µM) during the addition of 7.15 ppm ClO ^–^ and gallic acid; b) The relative absorbance ratio (A_580nm_/A_509nm_) of DMP-BTQ (10 µM) during the addition of 8.58 ppm ClO^-^ and gallic acid in ethanolic / PBS (10 mM, pH = 7.4). Insets: Visual color changes after each sequential addition of ClO^-^ and gallic acid under ambient light.

**
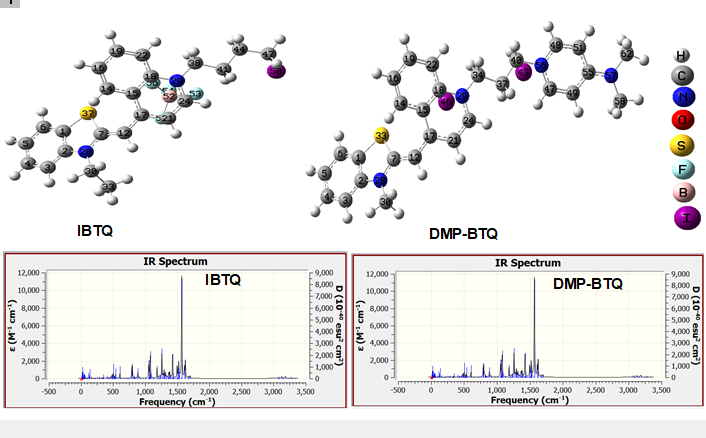
**

**Fig. S19**  (**Top**) IBTQ and DMP-BTQ optimized with labeled front, side, and (**bottom**) IR spectra in ethanol using the M06-2X/LANL2DZ measurement theory.


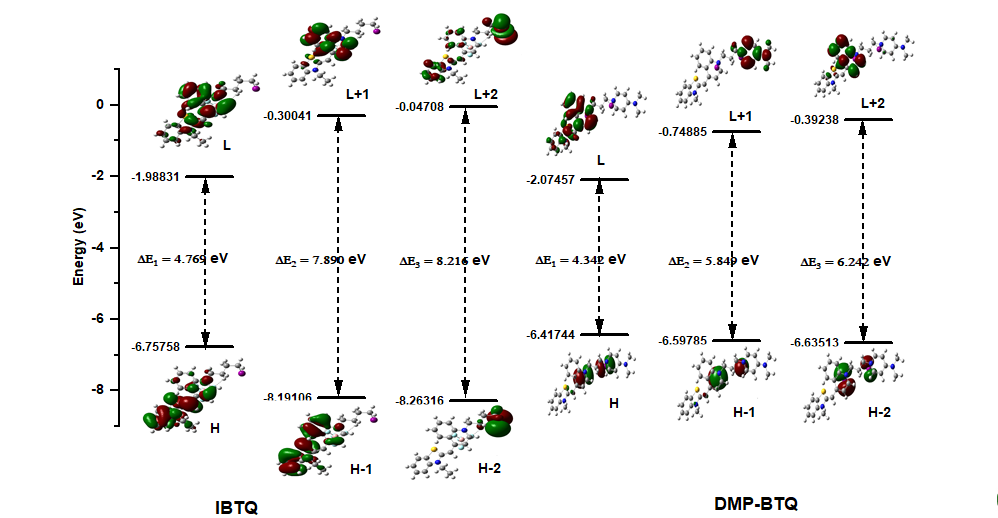


**Fig. S20** Graphic illustration of H/L, H-1/L+1, and H-2/L+2 energies, molecule distribution, and energy gaps (ΔE) for IBTQ and DMP-BTQ.


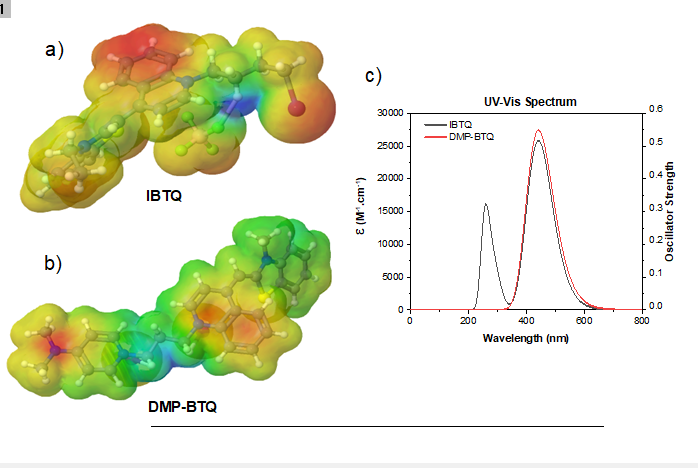


**Fig. S21** M06-2X/LANL2DZ electrostatic potential map (ESP) level of theory for **a)** IBTQ and **b)** DMP-BTQ. **c)** The absorption spectra estimated for both IBTQ and DMP-BTQ using the TD/ M06-2X/LANL2DZ technique.


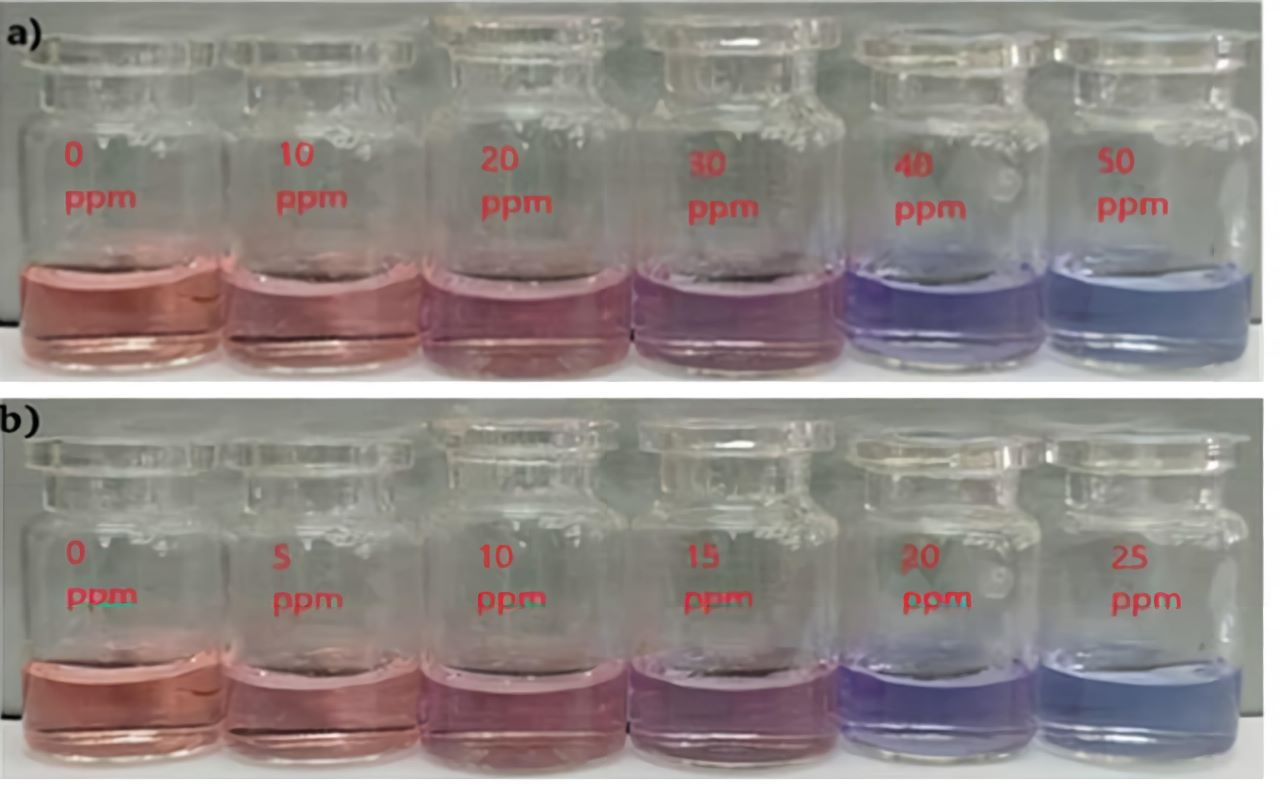


**Fig. S22** IBTQ (15 µM) **(a)** and DMP-BTQ (10 µM) **(b)** color change upon varying ClO^-^ concentrations.


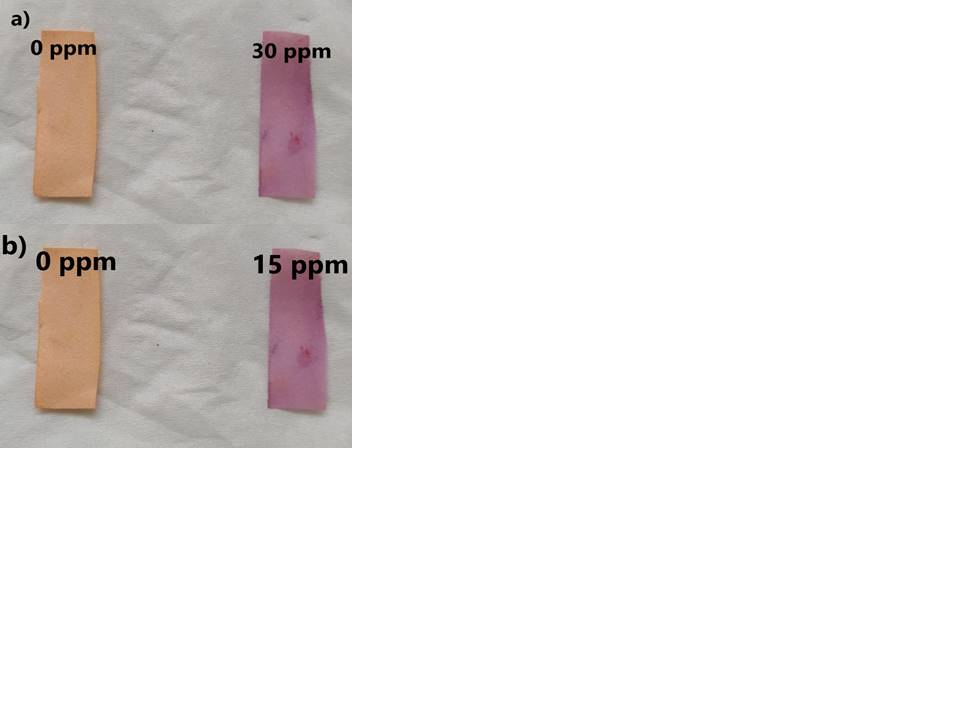


**Fig. S23** Photographs of a test paper-strip for ClO^−^ detection using **a**) IBTQ with 0 and 30 ppm ClO^-^ and **b**) DMP-BTQ with 0 and 15 ppm ClO^-^.


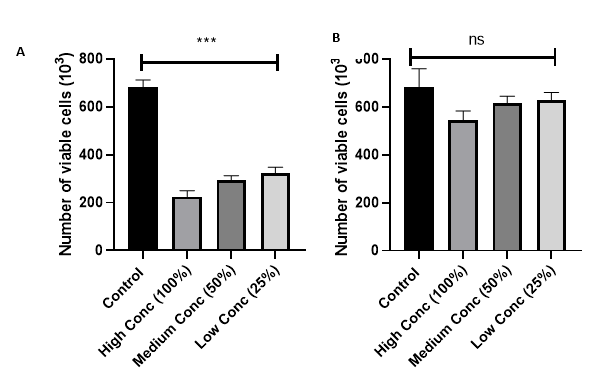


**Fig. S24 PBMCs viability after treatment with two sensors**: Number of viable cells after treating with IBTQ (A) DMP-BTQ (B). Cells were treated with (High (100%), Medium (50%), and low (25%) overnight, harvested, washed, and then counted***p value ≤0.001

**Table S1 Experimental (based on UV-Vis. spectrophotometer signal) versus calculated LOD values of IBTQ and DMP-BTQ.**

|  | Calculated LOD | Experimental LOD |
| --- | --- | --- |
| IBTQ | 13.92 ppm | 14 ppm |
| DMP-BTQ | 0.127 ppm | 3 ppm |

**Table S2.** **Comparison of LOD values of IBTQ and DMP-BTQ with those of some previously reported HOCl/OCl^-^ probes in literature.**

| **Method** | **Sensing**  **method** | **LOD**  (μM) | **Linear range**  (μM) | **Ref** |
| --- | --- | --- | --- | --- |
| DPD reagent (APHA and AWA, 2017) | Colorimetric | 5.0 | 0.01–5 | ^11^ |
| Phenothiazine-based ratiometric fluorescent probe LD-Lyso | Fluorometric | 8.74 | 0–22 | ^12^ |
| Cycometalated iridium (III) complex-based sensor | Fluorometric | 1.378 | (0–12 equivalents) | ^13^ |
| Dual-ligand luminol-Tb-GMP coordination polymer nanoparticles | Fluorometric | 0.14 | 2–18 | ^14^ |
| Carbazole-heptamethine cyanine dyad | Colorimetric | 0.014 | (0–10 equivalents) | ^15^ |
| Bithiophene-Meldrum's acid based chemosensor 2TM | Colorimetric | 0.048 | (0–2  equivalents) | ^16^ |
| Cyanine-based spiropyran fluorogenic probe | Fluorometric | 3.0 | (0–40 equivalents) | ^17^ |
| Green-emitting carbon dots (G-CDs) | Colorimetric | 1.82 | 10–150 | ^18^ |
| Microbial potentiometric sensor | Potentiometric | 2.7 | 0–2.7 | ^19^ |
| Commercial platinum Electrodes | Amperometry | 10.0 | 0.1–10 | ^20^ |
| Coumarin-salicylic hydrazide Schiff base (CMSH) | Fluorometric | 0.128 | 0–80 | ^21^ |
| IBTQ | Colorimetric  QCM | 13.92 ppm  0.06 ppm | )4.3–47.18) ppm  (0.42–1.53) ppm | The Current work |
| DMP-BTQ | Colorimetric  QCM | 0.127 ppm  0.045 ppm | (3.03–24.31) ppm  (0.142–0.68) ppm | The Current work |

**Table S3 Determination of ClO^-^ in real water samples using IBTQ and DMP-BTQ** **versus DPD using UV-Vis. absorption.**

|  | | **DPD method** | | **IBTQ** | **DMP-BTQ** |
| --- | --- | --- | --- | --- | --- |
| **Real water sample** | **ClO^-^ spiked (ppm)** | | **ClO^-^ found.**  **(ppm)_a_** | **ClO^-^ found.**  **(ppm)_a_** | **ClO^-^ found.**  **(ppm)_a_** |
| KZ tap water | 4.22 | | 4.23 ± 0.03 | 4.23 ± 0.03 | 4.22 ± 0.20 |
| Tanta tap water | 4.22 | | 4.22 ± 0.03 | 4.23 ± 0.02 | 4.20 ± 0.1 |
| Distilled water | 4.22 | | 4.23 ± 0.01 | 4.21 ± 0.05 | 4.23 ± 0.03 |
| Bottled water | 4.22 | | 4.23 ± 0.01 | 4.22 ± 0.06 | 4.21 ± 0.08 |

^a^ Mean ± standard deviation (n = 3)

**Table S4** Optimized structural parameters (bond length in Å, bond angle (^o^), and dihedral angle (^o^) computed for IBTQ and DMP-BTQ. Refer to Fig. S19 for labeling instructions.

| Compound | IBTQ |  | DMP-BTQ |
| --- | --- | --- | --- |
| Designation | Values | Designation | Values |
| C1-C6 | 1.392 | C1-C6 | 1.393 |
| C1-S37 | 1.814 | C1-S33 | 1.814 |
| C12-C17 | 1.442 | C12-C17 | 1.442 |
| C21-C24 | 1.388 | C21-C24 | 1.387 |
| N29-C38 | 1.491 | N29-C34 | 1.487 |
| C47-I50 | 2.195 | C55-N57 | 1.350 |
| C1-C6-C5 | 118.129 | C1-C6-C5 | 118.128 |
| C21-C17-C15 | 116.578 | C21-C17-C15 | 116.350 |
| C19-C22-C18 | 119.922 | C19-C22-C18 | 119.911 |
| C7-C12-C17-C15 | 42.803 | C7-C12-C17-C15 | 40.625 |
| C12-C17-C15-C14 | 13.589 | C12-C17-C15-C14 | 14.237 |
| C16-C14-C15-C17 | 178.025 | C16-C14-C15-C17 | 178.473 |

**Table S5 Quantum chemical characteristics for IBTQ and DMP-BTQ in ethanol, E_H_, E_L_, energy gap (E_g_), dipole moment (μ), electronegativity (χ), chemical potential (ρ), and chemical hardness (η).**

| MSs | E_H_ (eV) | E_L_ (eV) | E_g_(eV) | μ (D) | χ (eV) | ρ (eV) | η (eV) |
| --- | --- | --- | --- | --- | --- | --- | --- |
| IBTQ | -6.757 | -1.988 | 4.769 | 17.306 | 4.372 | -4.372 | 2.384 |
| DMP-BTQ | -6.417 | -2.074 | 4.342 | 31.028 | 4.245 | -4.245 | 2.171 |

**Table S6 Calculated electronic absorption parameters for IBTQ and DMP-BTQ.**

| TD-Computational | | | | | Exp. λ _abs._ (nm) |
| --- | --- | --- | --- | --- | --- |
| IBTQ | | | | |  |
| Excited state | Electronic transitions | ΔE (eV) | f | Coefficient | 505 |
| 1 | H-> L | 2.8043 (442.12nm) | 0.6383 | 0.6943 |  |
| 2 | H -> L+1  H-1-> L  H-4-> L | 4.232 (292.92nm) | 0.1312 | 0.16352  0.56122  0.3489 |  |
| 3 | H -> L+1  H-> L+2 | 4.382 (282.91 nm) | 0.0025 | 0.5655  0.1438 |  |
| DMP-BTQ | | | | |  |
| 1 | H -> L  H-1 -> L | 2.640 (469.52 nm) | 0.1707 | 0.26680  0.38830 | 509 |
| 2 | H -> L  H-1 -> L  H-3 -> L | 2.777 (446.39 nm) | 0.0406 | 0.29170  0.52736  0.15170 |  |
| 3 | H -> L  H-2 -> L  H-3 -> L | 2.8277 (438.46 nm) | 0.1170 | 0.14479  0.58347  0.10984 |  |

**Table S7** Selection of most influential second-order perturbation (E^2^) estimation of the hyper conjugative energies (kcal/mol) of IBTQ and DMP-BTQ molecular modeling structures.

| Donor | Acceptor | E^2^ (kcal/mol) | Donor | Acceptor | E^2^ (kcal/mol) |
| --- | --- | --- | --- | --- | --- |
|  |  | IBTQ |  |  | DMP-BTQ |
| πC1-C6 | π*C2-C3 | 30.31 | πC1-C2 | π*C3-C4 | 23.98 |
| πC1-C6 | π*C4-C5 | 24.11 | πC1-C2 | π*C5-C6 | 21.15 |
| πC2-C3 | π*C1-C6 | 27.11 | πC3-C4 | π*C1-C2 | 27.81 |
| πC2-C3 | π*C4-C5 | 30.87 | πC2-C3 | π*C4-C5 | 34.52 |
| πC4-C5 | π*C1-C6 | 33.90 | πC5-C6 | π*C3-C4 | 32.33 |
| πC4-C5 | π*C2-C3 | 28.55 | πC14-C16 | π*C19-C22 | 28.02 |
| πC14-C16 | π*C19-C22 | 28.06 | πC18-N29 | π*C21-C24 | 36.60 |
| πC18-N29 | π*C21-C24 | 37.28 | πC19-C22 | π*C14-C16 | 22.38 |
| πC19-C22 | π* C18-N29 | 45.34 | πC19-C22 | π* C18-N29 | 45.70 |
| LP (1) N28 | π*C2-C3 | 44.52 | LP (1) N28 | π*C1-C2 | 43.84 |
| LP (1) N28 | π*C7-C12 | 62.02 | LP (1) N28 | π*C7-C12 | 63.06 |

1 Alganzory, H. H., El-Sayed, W. A., Arief, M. H., Amine, M. S. & Ebeid, E.-Z. M. Microwave synthesis and fluorescence properties of homo- and heterodimeric monomethine cyanine dyes TOTO and their precursors. *Green Chemistry Letters and Reviews* **10**, 10-22, doi:10.1080/17518253.2016.1258088 (2017).

2 Alganzory, H. H., Arief, M., Amine, M. & Ebeid, E. Microwave-assisted solvent-free synthesis and fluorescence spectral characteristics of some monomethine cyanine dyes. *J. Chem. Pharm. Res* **6**, 143-161 (2014).

3 Ayad, M. M., Abdelghafar, M. E., Torad, N. L., Yamauchi, Y. & Amer, W. A. Green synthesis of carbon quantum dots toward highly sensitive detection of formaldehyde vapors using QCM sensor. *Chemosphere* **312**, 137031, doi:10.1016/j.chemosphere.2022.137031 (2023).

4 Amer, W. A. *et al.* Green synthesis of carbon quantum dots from purslane leaves for the detection of formaldehyde using quartz crystal microbalance. *Carbon* **179**, 159-171, doi:<https://doi.org/10.1016/j.carbon.2021.03.047> (2021).

5 AboAlhasan, A. A. *et al.* Enhanced energy transfer from diolefinic laser dyes to meso-tetrakis (4-sulfonatophenyl) porphyrin immobilized on silver nanoparticles: DFT, TD-DFT and spectroscopic studies. *Journal of Saudi Chemical Society* **26**, 101491 (2022).

6 Khalili, B., Mamaghani, M. & Bazdid-Vahdati, N. Structural design and physicochemical specifications exploring of the new di-cationic ionic liquids (D-ILs) composed of para-xylyl linked N-Methylimidazolium cation and various anions: a full M06–2X computational study. *Theoretical Chemistry Accounts* **141**, 3, doi:10.1007/s00214-021-02862-6 (2022).

7 Najwa, H. with The Imine and Some of its Derivatives via M06-2X Theory with Basis Set 6-31g(d) and Complete Basis Set. *Tishreen University Journal -Basic Sciences Series* **44**, 45-55 (2022).

8 Ugurlu, G. H., A. Investigation on Molecular Structure and Electronic Properties of Zinc (II) Complex with 2-acetylpyridinenicotinichydrazone Ligand *The Eurasia Proceedings of Science Technology Engineering and Mathematics* **20**, 58-65 . (2022).

9 Toy, M., Vural, H. & Şenöz, H. Synthesis, Spectroscopic (FT-IR, 1H NMR, and UV-Vis) and Nonlinear Optical Properties of a Novel 3-(p-Cyanophenyl)-5-(o, m, p-Iodophenyl)-1-Phenylformazan: Experimental and DFT Studies. *Polycyclic Aromatic Compounds*, 1-18, doi:10.1080/10406638.2022.2133903 (2022).

10 Abkari, A., Chaabane, I. & Guidara, K. DFT (B3LYP/LanL2DZ and B3LYP/6311G+(d,p)) comparative vibrational spectroscopic analysis of organic–inorganic compound bis(4-acetylanilinium) tetrachlorocuprate(II). *Physica E: Low-dimensional Systems and Nanostructures* **81**, 136-144, doi:<https://doi.org/10.1016/j.physe.2016.03.010> (2016).

11 Moberg, L. & Karlberg, B. An improved N,N′-diethyl-p-phenylenediamine (DPD) method for the determination of free chlorine based on multiple wavelength detection. *Analytica Chimica Acta* **407**, 127-133, doi:<https://doi.org/10.1016/S0003-2670(99)00780-1> (2000).

12 Liu, Q. *et al.* A water-soluble colorimetric and ratiometric fluorescent probe based on phenothiazine for the detection of hypochlorite ion. *Dyes and Pigments* **215**, 111194, doi:<https://doi.org/10.1016/j.dyepig.2023.111194> (2023).

13 Tian, T., Xu, S., Ru, Y., Zhang, D. & Pu, S. A red-emission iridium (Ⅲ) complex-based fluorescent probe with Schiff base structure for selection detection HOCl and its application in water sample. *Journal of Organometallic Chemistry* **976**, 122351, doi:<https://doi.org/10.1016/j.jorganchem.2022.122351> (2022).

14 Han, J. *et al.* Visualizing stimulus-responsive dual-ligand fluorescent probes for hypochlorite: A novel strategy for real application in tap water. *Luminescence* **38**, 722-728, doi:<https://doi.org/10.1002/bio.4500> (2023).

15 Pan, H. *et al.* A dual-function colorimetric probe based on Carbazole-Cyanine dyad for highly sensitive recognition of cyanide and hypochlorous acid in aqueous media. *Talanta* **202**, 329-335, doi:<https://doi.org/10.1016/j.talanta.2019.05.009> (2019).

16 Zeng, Y.-N. *et al.* Dual-emissive metal–organic framework: a novel turn-on and ratiometric fluorescent sensor for highly efficient and specific detection of hypochlorite. *Dalton Transactions* **49**, 9680-9687, doi:10.1039/D0DT02041A (2020).

17 Samanta, S., Halder, S., Manna, U. & Das, G. Specific detection of hypochlorite: a cyanine based turn-on fluorescent sensor. *Journal of Chemical Sciences* **131**, 36, doi:10.1007/s12039-019-1612-y (2019).

18 Bu, Y. *et al.* Green-emitting carbon quantum dots as a dual-mode fluorescent and colorimetric sensor for hypochlorite. *Analytical and Bioanalytical Chemistry* **414**, 2651-2660, doi:10.1007/s00216-022-03901-2 (2022).

19 Saboe, D. *et al.* Measurement of free chlorine levels in water using potentiometric responses of biofilms and applications for monitoring and managing the quality of potable water. *Science of The Total Environment* **766**, 144424, doi:<https://doi.org/10.1016/j.scitotenv.2020.144424> (2021).

20 Wilson, R. E., Stoianov, I. & O’Hare, D. Continuous chlorine detection in drinking water and a review of new detection methods. *Johnson Matthey Technology Review* **63**, 103-118 (2019).

21 He, X. *et al.* Ratiometric and colorimetric fluorescent probe for hypochlorite monitor and application for bioimaging in living cells, bacteria and zebrafish. *Journal of Hazardous Materials* **388**, 122029, doi:<https://doi.org/10.1016/j.jhazmat.2020.122029> (2020).
